# A Preliminary Data-Driven Approach for Classifying Knee Instability During Subject-Specific Exercise-Based Game with Squat Motions

**DOI:** 10.3390/s25196074

**Published:** 2025-10-02

**Authors:** Priyanka Ramasamy, Poongavanam Palani, Gunarajulu Renganathan, Koji Shimatani, Asokan Thondiyath, Yuichi Kurita

**Affiliations:** 1 Graduate School of Advanced Science and Engineering, Hiroshima University, Hiroshima 739-8527, Japan; ykurita@hiroshima-u.ac.jp; 2 Department of Engineering Design, Indian Institute of Technology Madras, Tamil Nadu 600-036, India; poongavanambme@gmail.com (P.P.); asok@iitm.ac.in (A.T.); 3 Cyber Physical Systems, Graduate School of Innovation and Practice for Smart Society, Hiroshima University, Hiroshima 739-8529, Japan; gunarajulu@hiroshima-u.ac.jp; 4 Department of Physical Therapy, Prefectural University of Hiroshima, Hiroshima 723-0053, Japan; shimatani@pu-hiroshima.ac.jp

**Keywords:** knee instability, long short-term memory, support vector machine, exergames, squats

## Abstract

Lower limb functional degeneration has become prevalent, notably reducing the core strength that drives motor control. Squats are frequently used in lower limb training, improving overall muscle strength. However, performing continuously with improper techniques can lead to dynamic knee instability. It worsens with little to no motivation to perform these power training motions. Hence, it is crucial to have a gaming-based exercise tracking system to adaptively enhance the user experience without causing injury or falls. In this work, 28 healthy subjects performed exergame-based squat training, and dynamic kinematic features were recorded. The five features acquired from a depth camera-based inertial measurement unit (IMU) (1—Knee shakiness, 2—Knee distance, and 3—Squat depth) and an Anima forceplate sensor (4—Sway velocity and 5—Sway area) were assessed using a Spearman correlation coefficient-based feature selection method. An input vector that defines knee instability is used to train and test the Long Short-Term Memory (LSTM) and Support Vector Machine (SVM) models for binary classification. The results showed that knee instability events can be successfully classified and achieved a high accuracy of 96% in both models with sets 1, 2, 3, 4, and 5 and 1, 2, and 3. The feature selection results indicate that the LSTM network with the proposed combination of input features from multimodal sensors can successfully perform real-time tracking of knee instability. Furthermore, the findings demonstrate that this multimodal approach yields improved classifier performance with enhanced accuracy compared to using features from a single modality during lower limb therapy.

## 1. Introduction

Functional deficiencies are key contributors to physical health deterioration and frequently coexist with various comorbidities, such as joint rigidity, neuromuscular diseases, sensorimotor degeneration, and chronic diseases. They are widely observed in all age groups, with a prevalence of up to 15% in middle-aged individuals and precisely 25% in older adults [1]. The clinical relevance of this decline in physical conditions includes 3% to 8% of visits to clinics and rehabilitation centers. However, several training regimens for people with functional degeneration strongly emphasize organized physical training to avoid frailty conditions and improve mobility at an early stage. These training regimens include exercise-based therapies like sensorimotor reconditioning and musculoskeletal training for those experiencing declining mobility and reduced progress in rehabilitation outcomes. The training also necessitates continuous monitoring for around 3 to 6 months to evaluate the performance and adaptation based on task-specific motions.

To address the challenges of adaptation and user engagement with consistent monitoring, several exercise-based games have been developed to improve mental well-being and physical performance, facilitating recovery and restoring mobility. A simulated model was proposed that establishes rigorous training and promotes an on-body feedback system at an early stage, resulting in increased accessibility and reduced barriers to exercise environments [2]. In addition, exergaming models also focus on individual adaptation and are extensively evaluated using a target-based approach to respect physical and cognitive abilities [3]. Other priorities include implementing a safe and unsupervised platform that provides the automated adaptability necessary to reduce the demand for physical therapists [4]. Individual adaptation also influences game preferences, particularly with older adults, who tend to choose more flexible and interactive games with the integration of entertaining game mechanics. This organic conception must be investigated by evaluating the motivation and effectiveness factors during exergame development [5,6]. Squats are widely recognized as a primary form of resistance and strength training activity, which can be employed for effective lower limb monitoring. The movement of the knees and hips plays a crucial role in influencing trunk orientation [7]. Among the various squat forms, the parallel squats (knee angle between 70° and 100°) and deep squats (knee angle >100°) are the most commonly practised in strength training routines. When the knee angle reaches higher than 100° due to improper movement execution, repetitive strain, or excessive loading, it can lead to compromised knee stability [8]. Furthermore, the condition causes injuries to passive restraints like the ACL (anterior cruciate ligament) or PCL (posterior cruciate ligament), combined with impaired neuromuscular control (patellofemoral pain syndrome) and early-onset osteoarthritis [9]. The compromised stability increases the development of involuntary, oscillatory movements around the knee joint, which is commonly referred to as knee instability. Knee instability (KI) is a visible indicator of dynamic instability for assessing joint control, proprioceptive function, and risk of injury in clinical settings [10]. Consequently, a strong demand exists for designing a system that effectively monitors knee instability indicators. Few studies on detecting knee instability during single-leg squats exhibit a low knee flexion and a slow increase in valgus velocity [11].

**Figure 1:**
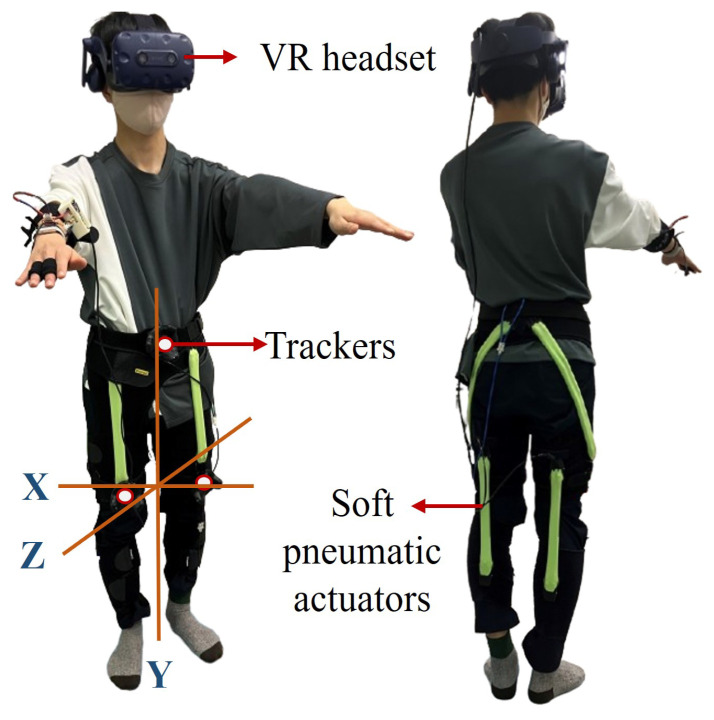
User performing a squat in a virtual reality (VR) exergaming scene wearing controllers and a suit with pneumatic gel muscle (PGM)-based actuators.

Lower limb abnormalities can persist or worsen without targeted motor assist and balance control while performing core muscle training, such as squats, making it challenging to tailor training intensity to each individual’s needs. Researchers have developed robotics-based adaptive training systems to overcome these issues and improve lower limb strength while enhancing postural control. Previous studies demonstrated the potential of pneumatic gel muscle (PGM) suits, as shown in Figure 1, which were integrated with a gaming-based protocol that allows users to perform optimized squat motions. This module promotes adaptation and motivation to posture by evaluating the lower extremity on joint characteristics [12] and muscle activity patterns [13]. The combined virtual reality (VR) and force feedback module investigates the performance effects of providing illusive movements to climb stairs in virtual space, improving lower limb rehabilitation [14]. While these studies highlight the promising method of combining PGM-based systems with VR feedback for exergaming rehabilitation, their approaches remain limited to the lack of data-driven methods for time series estimation.

The primary objective of this study is to utilize a novel approach of an exercise game that integrates multimodal features from IMU sensors and a force plate, coupled with data-driven models of LSTM and SVM, for classifying knee instability. This data-driven approach addresses the inconsistencies in previous studies and provides a robust pathway towards personalized and adaptive rehabilitation interventions. The structure of this paper is organized as follows. Section 2, presents the related studies, with Table 1 providing a detailed overview of state-of-the-art approaches that have utilized this evaluation framework to assess knee models with various machine learning methods. Section 3 presents the experimental protocol, detailing the design and execution of controlled trials to analyze knee and center of pressure (CoP) parameters. This section also describes the data collection and feature selection to identify practical features and classification models considered. Section 4 presents the results and detailed discussion, followed by conclusions with potential implications and future work in Section 5.

## 2. Related Work

AI has played a significant role in real-time assessment for assistive and rehabilitative robotics. These systems aim to support the monitoring and adaptive control of exercise intensity, thereby ensuring personalized rehabilitation plans [29]. For example, Tadayon et al. proposed an artificial neural network (ANN) model to predict the progression of locomotive disorders using the joint tracking data from exergames and lower limb exercises. Their model linked performance characteristics to locomotive risk and utilized the predicted accuracy to train the target groups accordingly [30]. Similarly, Cary et al. utilized ANNs and Kinect-based posture and gesture recognition, aiding therapists in tracking patient health data for physiotherapy evaluations [31]. Vonstand et al. used a comparative study of multiple machine learning models, such as the multilayer perceptron, random forest, SVM, and K-nearest neighbor models, to assess weight-shifting exercise using full-body data [32]. Additional work has focused on features, such as joint angles and knee surface deformation, to evaluate knee functionality using stretch sensors [33]. Zhengtao et al. analyzed the noisy and denoisy signal from surface electromyography (EMG) sensors to classify knee joint vibrations using SVM [34], while Vijayavargiya et al. used five machine learning models to detect the knee abnormalities using sEMG features [20]. These methods enable non-invasive knee monitoring but often rely on unimodal inputs, limiting their accuracy and generalizability.

To address this, some investigations have employed multimodal parameters of muscle forces, knee joint coordinates, and foot pressure for locomotion recognition [35] and motion prediction [22]. Oh et al. developed a classifier based on joint angles and foot pressure, captured via Kinect-based IMUs and Wii balance boards, to recognize squat postural variations [23]. While these studies incorporate multiple input modalities, they often use conventional machine learning methods with moderate classification accuracy and limited robustness to biomechanical variations such as knee instability.

Few works examined the application of deep learning models like LSTM for knee monitoring during gait and squat-based motions, often accompanied by comparative analysis with traditional classifiers. For instance, Narayan et al. employed a combination of knee joint angles from goniometer sensors and EMG data to evaluate gait health using LSTM models trained on two different types of optimizers (SGD and ADAM), achieving moderate accuracy [25]. While utilizing single-modality inputs, Vijayvargiya et al. extended their previous study by using a CNN-LSTM hybrid model. This approach effectively captured both spatial and temporal patterns in sEMG signals during sitting, standing, and walking movements [24]. Mohsen et al. employed LSTM to classify gait abnormalities using joint features obtained from the Kinect system, achieving lower accuracies [26]. Felix et al. evaluated motion errors in a VR-based coaching system using squat techniques with an avatar training, where the Conv-SLTM among three classifiers showed moderate performance with an accuracy of around 80% [27]. These findings highlight that while LSTM models are frequently applied, their performance remains below optimal, typically achieving moderate classification accuracy.

Our proposed study presents preliminary results on the use of exergaming interfaces that incorporate combined sensory inputs from camera and force sensor modalities. This approach also facilitates an improved classification of mobility limitations by enabling robust real-time interactions and movement monitoring. Advanced models like LSTM networks have the potential to enhance rehabilitation results with enhanced accuracy by systematically capturing postural deviations as well as temporal dynamics.

## 3. Materials and Methods

**Figure 2:**
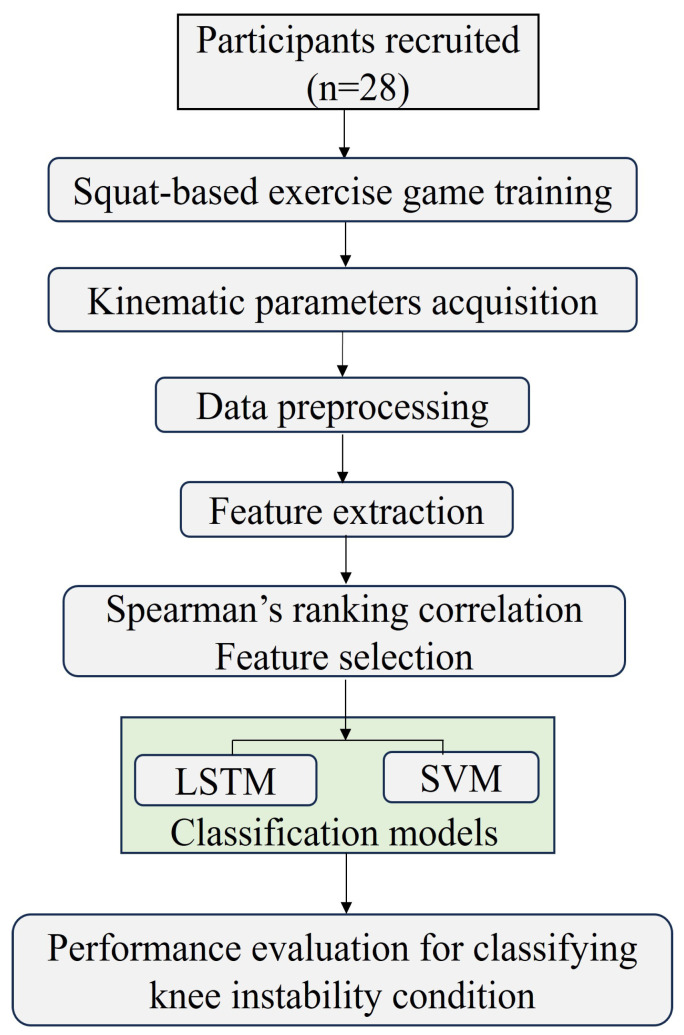
Illustration describes the workflow of squat-exergame with knee instability classification technique.

In this section, we discuss gameplay development and the system requirements for acquiring and extracting significant features for classification inputs. In addition, we describe the types of feature selection and classification models best suited for estimating performance in classifying knee instability events. The flow of the tasks involved in the methodology is illustrated in Figure 2.

### 3.1. Gameplay Configuration

**Figure 3:**
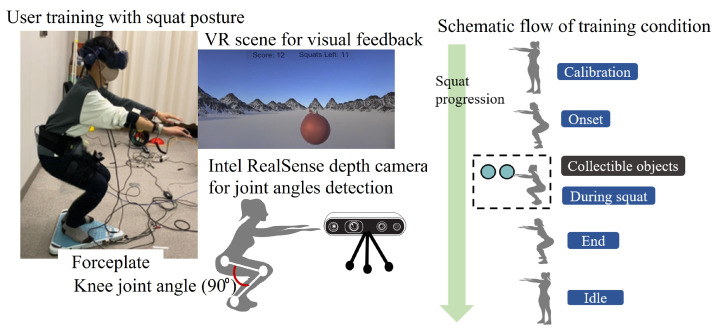
Exercise-based gaming technique with the schematic of squat phases.

Our squat exergaming module [13], built with a Unity-based interface, offers a reliable and immersive virtual environment. The system employs an HTC Vive headset to interact with the user, enabling calibration procedures such as measuring the user’s height from the ground and defining the distinct phases of the squat motion. To synchronize the user’s motion with that of the gaming object, we attached Vive trackers to the torso and both knees that constantly track the trajectory to align with the position and orientation of the headset. The schematic algorithm shown in Figure 3 provides an overview of the squat phases to determine the exergaming conditions. The system then integrates a depth camera (Intel RealSense D435i, Intel Corporation, Santa Clara, CA, USA) to monitor the user’s joints, capturing data at approximately 60 frames per second (fps) to measure the lateral displacement of the hip and knee joints. The measured velocity data acquired from joint coordinates were then used to estimate the knee metrics. We also used a portable gravicorder (BW-6000+MD, ANIMA, Tokyo, Japan) to assess the projection area of the centre of gravity, which facilitated measuring kinetic changes at the body’s center of pressure during squatting. The data are sampled at a frequency of 50 Hz. Figure 3 depicts the squat exercise gaming configuration and squat progression flow with different phases.

### 3.2. Participants

This study was performed with twenty-eight healthy young subjects from the Prefectural University of Hiroshima, Mihara campus. The users include twenty-two males and six females with a mean age of 20.1 ± 0.8 years, a mean height of 169.6 ± 7.9 cm, and a mean weight of 59.6 ± 9 kg. The participants were selected based on conditions with no medical history of lower limb impairments, particularly knee pain or discomfort. Moreover, they were instructed not to participate in power training until the user study was completed, as squatting is already an intensive training gesture. This study was approved by the Institutional Review Board (C-342) research ethics committee at Hiroshima University Hospital to conduct the study while adhering to the Declaration of Helsinki’s ethical guidelines. All subjects were informed of the experimental conditions and consented to them before the commencement of the user study.

### 3.3. Experimental Protocol

The exergaming scenario is developed with the goal of collecting the game objects with a headset, which are spheres that appear on the path of motion. This condition allows the users to perform self-adaptive squats with the range of each squat depth from 0° to 110° knee flexion according to the body dimensions, such as the height of the user [36]. The trackers were attached using Velcro tapes for secure fastening while the user was standing on the gravicorder. The participants were asked to perform a general warm-up or stretch routine before the study began to avoid any physical injury or discomfort during the training. The gaming scene starts with two squat trials for calibrating after the user’s comfort is validated. The subjects engaged in a structured squat exercise conducted multiple times per week, as shown in Figure 3. In addition, subjects are given a 90-second rest interval to prevent fatigue or falling.

As shown in the schematic flow, the user starts to descend during the ‘Onset’ phase, trying to flex their knees parallel to the ground. The condition will enable the user to focus on the collectible spheres coming their way. After that, the user will tend to hold the parallel squat position (knee flexion angle = 90°), enabling collision for accumulating the gaming object ’during squat’ phase. The user will ascend back to the upright standing position during the ’End’ phase. We also made it possible for users to view their scores and squat counts, helping maintain motivation and engagement. The Unity-based algorithm for formulating the squat phase detection was illustrated in our previous study [13].

### 3.4. Data Collection and Preprocessing

We used dedicated systems to acquire camera-driven knee indicators and the force plate-driven CoP parameters in this study. Initially, the raw data of lower limb joint coordinates from the depth camera were acquired. Among them, the knee joint coordinates were used to formulate knee features. In contrast, dynamic data of CoP features were used to calculate sway-based metrics. All streams were timestamped and synchronized by linear interpolation to a common sampling grid. Though both systems synchronized with data recording, excluding the calibration trails, there are some outliers with a few seconds difference in the start times. We removed outliers using a digital 4th-order low-pass Butterworth filter with a cut-off of 5 Hz from MATLAB (R2024a). The segmentation was performed in MATLAB to ensure reproducibility. Spurious detections were rejected, and fixed-length windows were extracted for each valid squat to support consistent feature computation and labeling. The segmentation can also perform standardization to downstream and consume uniform, per-cycle vectors. The knee parameters of knee shakiness (KS), knee distance (KD), and squat depth (SD) were estimated using the formulations (1) to (3) described in previous work [12]. The CoP parameters of sway velocity (SV) and sway area (SA) were then calculated using Equations (4) and (5) as illustrated in [37].

(1) $$\begin{array}{c} \begin{array}{cc} KS & =V_{l}\left(t\right)+V_{r}\left(t\right)\\ V_{l}\left(t\right) & =\left|\frac{x_{lk}\left(t\right)-x_{lk}\left(t-1\right)}{\Delta t}\right|\\ V_{r}\left(t\right) & =\left|\frac{x_{rk}\left(t\right)-x_{rk}\left(t-1\right)}{\Delta t}\right| \end{array} \end{array}$$

(2) $$KD=\left|x_{lk}-x_{rk}\right|$$

(3) $$SD=\left|y_{t_{3,\mathrm{peak}}}-y_{t_{1}}\right|$$

In Equations (1)–(3), $V_{l}\left(t\right)$ and $V_{r}\left(t\right)$ represent the velocities, *t* is the time, while $x_{lk}$ and $x_{rk}$ are the knee positions in the sagittal axis of both knees, respectively.

In Equation (3), $y_{t_{3,\mathrm{peak}}}$ is the minimum torso displacement and $y_{t_{1}}$ is the torso position in the transverse axis, respectively.

(4) $$\begin{array}{cc} \mathrm{SV}=\; & \left(\sqrt{{\left({\mathrm{CoP}}_{y}\left(t+1\right)-{\mathrm{CoP}}_{y}\left(t\right)\right)}^{2}}\right.+\\ \; & \left.\sqrt{{\left({\mathrm{CoP}}_{x}\left(t+1\right)-{\mathrm{CoP}}_{x}\left(t\right)\right)}^{2}}\right)\cdot \Delta t \end{array}$$

(5) $$\begin{array}{c} \mathrm{SA}=\;\frac{|{\mathrm{CoP}}_{x}\left(t+1\right)\cdot {\mathrm{CoP}}_{y}\left(t\right)-{\mathrm{CoP}}_{x}\left(t\right)\cdot {\mathrm{CoP}}_{y}\left(t+1\right)|}{2\cdot \Delta t} \end{array}$$

In derivatives (4) and (5), ${CoP}_{x}$ and ${CoP}_{y}$ are the displacement coordinates in the medio-lateral (ML) and anteroposterior (AP) directions.

Inter-subject normalization is performed on the features to ensure variability while maintaining subject-specific biomechanics. Initially, the inter-squat normalization is performed across the squat cycles. Also, the intra-subject normalization is performed using global min-max values, which are time-normalized to 20 time samples across all subjects. We aimed to establish labeling between knee instability (KI) and non-knee instability (NKI) conditions based on the KS factor, as illustrated in [12,38]. The labeling is performed with a two-class classification after the trial, which indicates NKI with a score of ‘0’ if KS< 0.03 m/s, and KI is shown with the score of ‘1’ if KS≥ 0.03 m/s.

### 3.5. Feature Selection

Initial analysis considered around 14 parameters, including joint angles (knee and torso) in the sagittal, transverse, and frontal axes of squat motions, along with dynamic data of CoP variables. To avoid redundancy, it is crucial to select the most appropriate feature ranking models that help in dimensional simplification. Since our study utilizes kinematic features, we considered using a correlation-based feature selection model that best identifies and selects features across sequential time series data supporting phase-dependent analysis of squat patterns. Correlation-based feature selection was performed using Spearman’s rank correlation coefficient, which determines the nonlinearity between the intensity and direction of the considered features [39]. Each extracted knee parameter was ranked based on its correlation strength with the instability labels. This will ensure that the analysis prioritizes parameters that show the strongest consistent association with knee instability events. ρ and ϕ are the two coefficients that represent the Spearman ranking-based correlation.

The selected features are then rearranged to be fed into the machine learning (ML) models: (1) LSTM NN and (2) SVM. The models are trained with the 3639 squat data, with 3405 squats reflecting NKI and 234 squats reflecting KI.

#### 3.5.1. Long Short-Term Memory Neural Network (LSTM)

**Figure 4:**
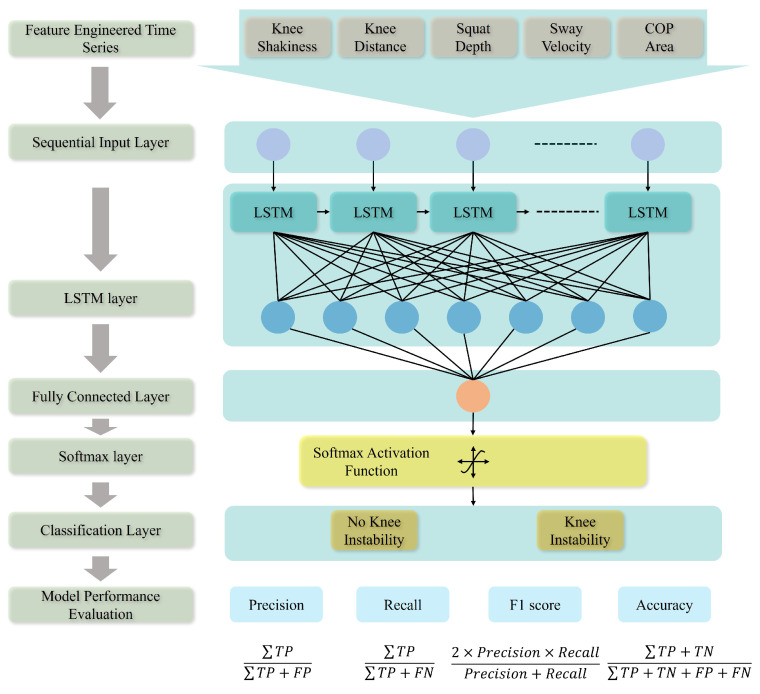
Illustration of the LSTM neural network architecture depicting the process from feature set selection to performance metrics estimation.

The recurrent neural network (RNN) is an extended version of a feed-forward neural network with three layers, such as an input layer *U*, a hidden layer *H*, and an output layer *V*. The hidden layer provides input from previous time steps, thus allowing short-term memory for the network. This memory dependencies property makes the LSTM appropriate for supervised classification [40]. The LSTM neural network implemented in this study consists of layers as seen in Figure 4 and is explained in detail in the following subsections as to how the vanishing gradient problem in RNNs is addressed in the LSTM network [41].

##### Input Layer

The features selected are in time series $U_{t}$ in cell format for each squat event and have been given as sequential input to the input layer.

##### LSTM Layer

**Figure 5:**
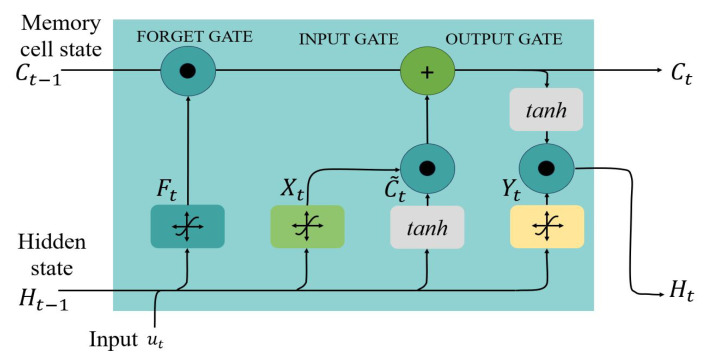
Structure of the LSTM cell with memory cell state $C_{t}$, hidden state $H_{t}$, and input $U_{t}$.

The core function of the network is an LSTM cell that regularizes the memory gradient issue with multiple gates, as shown in Figure 5. Assume there are *h* hidden units of batch size *k* and *m* inputs; then the input is defined as $U_{t}\epsilon R^{k\times m}$ with a previous time step hidden state as $H_{t-1}\epsilon R^{k\times h}$. Each gate in the memory cell at time step *t* can then be defined as the input gate $X_{t}\epsilon R^{k\times m}$, the forget gate $F_{t}\epsilon R^{k\times m}$, and the output gate $Y_{t}\epsilon R^{k\times m}$ with sigmoid functions that maintain the range of the resultant values to be (0,1). The $F_{t}$ decides how much memory has to be removed, while the $Y_{t}$ decides how much of the current output value should be modified. The results of the gates can be calculated as

(6) $$\begin{array}{c} \begin{array}{cc} \; & X_{t}=\sigma \left(U_{t}W_{ui}+H_{t-1}W_{hi}+b_{i}\right)\\ \; & F_{t}=\sigma \left(U_{t}W_{uf}+H_{t-1}W_{hf}+b_{f}\right)\\ \; & Y_{t}=\sigma \left(U_{t}W_{uo}+H_{t-1}W_{ho}+b_{o}\right) \end{array} \end{array}$$

where $W_{u}\epsilon R^{k\times m}$ is the weight vector and *b* is the bias. In addition to this, an input node ${\tilde{C}}_{t}\epsilon R^{k\times m}$ with a tanh activation function for the input value decides how much memory should be added to the current state of the LSTM cell and maintains the range between (−1, 1). The input node ${\tilde{C}}_{t}$ at time step *t* can be defined as

(7) $$\begin{array}{c} \begin{array}{cc} \; & {\tilde{C}}_{t}=tanh\left(U_{t}W_{uc}+H_{t-1}W_{hc}+b_{c}\right)\\ \; & C_{t}=F_{t}⊙C_{t-1}+I_{t}⊙{\tilde{C}}_{t} \end{array} \end{array}$$

Having defined the gates and the input node, we can now define the update equation showing how the input gate governs the impact of new data using ${\tilde{C}}_{t}$ and how $F_{t}$ governs how much of the previous memory cell state ${\tilde{C}}_{t-1}$ can be retained. The updated Equation (7) thus helps solve the vanishing gradient problem. This allows the network to decide when to learn based on the subsequent inputs. For example, if the output of $F_{t}$ is 1, then $I_{t}$ is 0, which results in ${\tilde{C}}_{t-1}$ being a constant. The output of the memory cell is the hidden state $H_{t}$ obtained using Equation (8), ensuring the $H_{t}$ range to be (−1, 1)

(8) $$H_{t}=Y_{t}⊙tanh\left(C_{t}\right)$$

When $Y_{t}$ is 0 or close to 0, the current memory will not affect or have minimal effect on the rest of the layers, respectively. A value close to 1 indicates that there will be an impact on the cell memory of the subsequent time steps. The output of the LSTM layer is given by

(9) $$\begin{array}{c} \hat{O_{t}}=\sigma \left(W_{ho}H_{t}+b_{o}\right) \end{array}$$

The fully connected layer receives input from the LSTM layer and transforms it based on the weight vector and bias, as shown in Equation (9). It provides a direct connection for each input node to the output node. The softmax layer consists of a sigmoid function σ(.) as in Equation (9), providing a probability value that helps the next layer decide in which class the input sequence can be categorized.

##### Classification Layer

Based on the values received from the softmax layer, the classification layer provides a binary output of whether the class is KI or NKI.

#### 3.5.2. Support Vector Machine (SVM)

In this study, SVM is used to compare with the LSTM NN. The SVM algorithm is used in many classification and regression applications but is predominantly used in supervised classification [42,43]. It works on finding the hyperplane that can separate the categories under different labels, such as the KI and NKI. Initially, the multi-feature input data are transformed to a higher dimensional space using kernels, and the SVM contains four popular kernels, such as (1) linear kernel, (2) polynomial kernel, (3) Gaussian radial basis function kernel, and (4) the sigmoid kernel. Here, we used the radial basis function (RBF) to find the decision boundary using a Sequential Minimal Optimization (SMO) technique. The Gaussian radial basis function *K* can be defined as,

(10) $$\begin{array}{c} k\left(u_{1},u_{2}\right)=exp(-||{\left(u_{1}-u_{2}\right)}^{2}\left||\right) \end{array}$$

where $k(u_{1},u_{2})$ maps the input to space *S*. The radial basis function aids in mapping our nonlinear data to the decision space *S*.

## 4. Results and Discussion

The model training was conducted using a system with an Intel(R) Core(TM) i7-11800H CPU @2.30 GHz and 64GB RAM. The two ML models were trained using distinct feature sets, and their effectiveness was analyzed and compared based on features using performance metrics.

### 4.1. Feature Based Comparison

**Figure 6:**
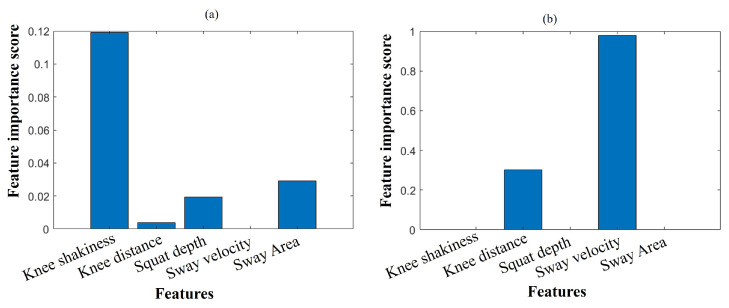
Feature ranking using Spearman correlation coefficient (**a**) ρ-indexed and (**b**) ϕ-indexed.

According to the correlation-based criterion, the ρ coefficient measures the monotonic relationship between the feature and target, which signifies continuous-time data. Figure 6a implies that KS, SD, and SA are the most significant features for classification. In addition, the ϕ coefficient is used to measure the non-linearity between the features, which implies a binary change in the data. The ϕ-indexed feature selection model strongly suggests that KD and SV are also the best input features for classification, as illustrated in Figure 6b. Hence, we have grouped the data by two modalities: camera-based and forceplate-based. KS, KD, and SD include a camera-based category with two continuous time series features and one binary feature. In contrast, the forceplate-based category comprises one continuous time series feature and one binary feature.

### 4.2. Performance Evaluation of LSTM and SVM Based on Feature Sets

In this study, a binary classification was performed to classify whether the squat event was tedious enough for the participants’ knees to be stable (KI) or not (NKI). The chosen LSTM NN is compared with the SVM for classification performance. The network was trained and tested using different feature sets, followed by an evaluation of its performance. Performance metrics are precision, recall, F1 score that are calculated according to correctly classified and incorrectly classified outputs as illustrated in Figure 4. Here, TP is the number of squat events that have been correctly classified as having KI, while *FP* is the number of events that have been incorrectly classified as having KI. On the other hand, the *TN* is the number of squat events without KI that are correctly classified as not having KI, and *FN* is the number of squat events that have KI but were incorrectly classified as without KI.

AUC (Area Under Curve)

**Figure 7:**
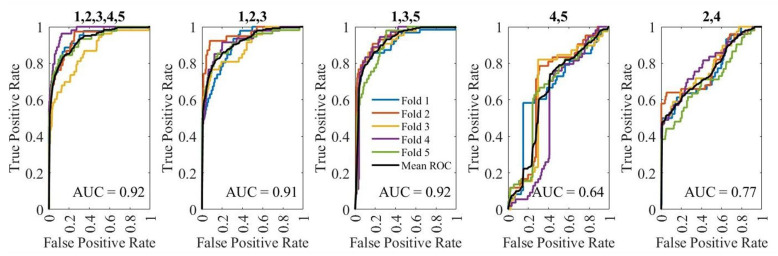
AUC for evaluating five feature sets combination for LSTM network with the feature combinations mentioned above each graph. (1—Knee shakiness, 2—Knee distance, and 3—Squat depth, 4—Sway velocity and 5—Sway area)).

**Figure 8:**
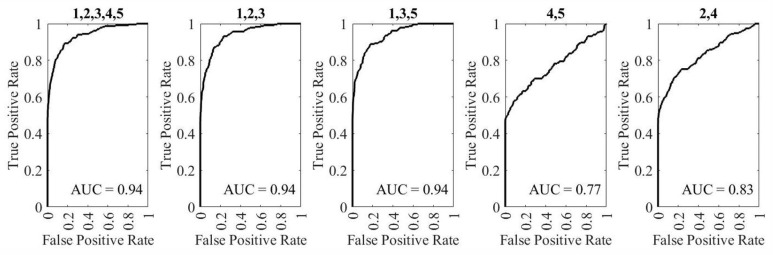
AUC for evaluating five feature sets combination for SVM network with the feature combinations mentioned above each graph. (1—Knee shakiness, 2—Knee distance, and 3—Squat depth, 4—Sway velocity and 5—Sway area)).

The AUC is calculated to show the goodness of network classification. The LSTM and SVM networks, the feature sets “1,2,3,4,5”, “1,2,3”, and “1,3,5” show better classification capabilities, as shown in Figure 7 and Figure 8.

Precision

The precision estimate is considered when a higher number of false positives (classifying KI as NKI) will result in system complexities. As shown in Table 2, the precision is higher for the feature sets “1,2,3,4,5” and “1,2,3”, while Table 3 shows no difference in the precision values for SVM.

Recall

It is important to note recall when a false negative (a KI event classified as NKI) is risky. In this sense, both “1,2,3,4,5” and “1,2,3” have better performance when compared to the rest of the sets for LSTM, and in the SVM classifier, there seems to be no significant recall value.

F1 score

An important metric for our study is the F1 score since the dataset used has more NKI than KI. This shows that the LSTM classifier with feature sets “1,2,3,4,5” and “1,2,3” shows good prominence.

**Figure 9:**
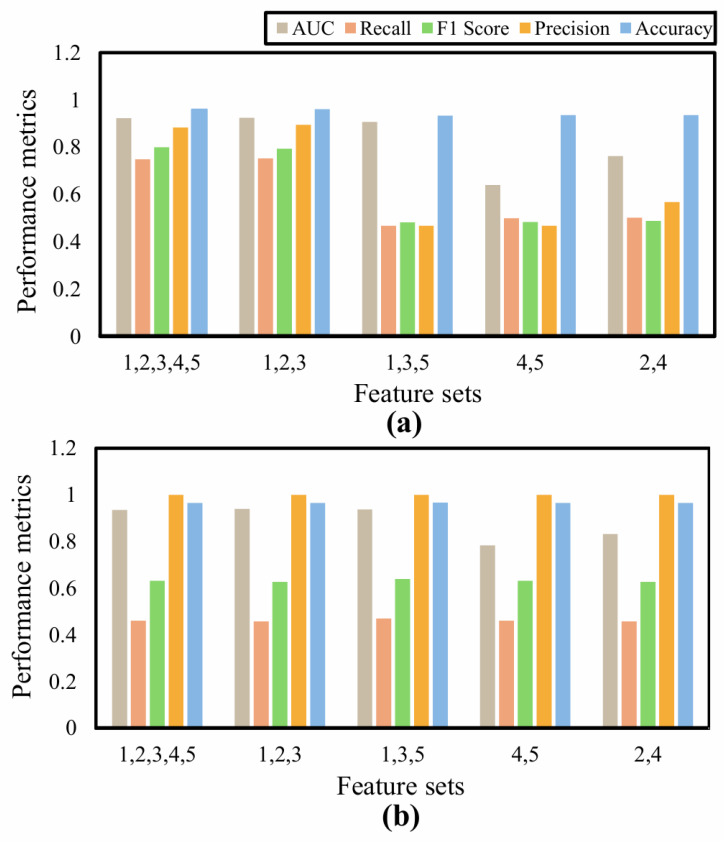
Classifier performance evaluation with feature-based comparison for (**a**) LSTM and (**b**) SVM.

There exists a general weakness for the SVM in the case of unbalanced datasets [44] despite the potential advantages of the generalizability of the classifier. Although SVM has good accuracy for almost all the feature sets, it is clear from the other performance metrics that LSTM performs better for the binary classification of the squat event during exergames. Also, a comparison of the performance metrics of the feature sets indicates that the “1,2,3,4,5” and “1,2,3” sets are best suited for the binary classification under consideration, as shown in Figure 9.

This shows that the characteristics describe instantaneous changes in knee position and stability when performing the squat; these are crucial factors to be considered for robust classification strategies.

## 5. Conclusions

In most cases, patients with knee-related impairments undergo conventional exercises at rehabilitation centers to regain stability and functional movement. Recent advancement in exercise-based games and wearable sensors provides patients with an engaging approach to train and improve their physical activities. However, the reliable detection of knee instability during such exercises remains a significant challenge. Current approaches often rely on manual assessment or limited features, making it complex to capture critical dynamics of instability across different phases of movements.

Our work reports an exercise-based game approach that integrates data-driven automated monitoring of knee instability conditions, using reliable and functional parameters suitable for real-world settings. Our approach addressed the challenge by achieving the goal of choosing the best feature combinations obtained from multiple modalities and applying a machine learning model to detect the dynamic change of knee kinematic features during squats. Additionally, the feature selection and classification results highlighted the importance of kinematic features when analyzing the squat-based knee instability conditions.

The proposed classifier model can enhance the embodied interaction and development of intelligent physiotherapy solutions. This can be further integrated with the exergame scene by adding rewards for good squats and penalties for bad squats, thus having a quantitative measure for improvement. In the future, we intend to apply this model with soft robotic interfaces that provide either haptic or vibrotactile feedback to investigate the efficacy in reducing the progression of knee instability events. This high-accuracy-based classifier module integration with soft wearable systems can significantly reduce the requirement for repeated assessment and improve adherence to the therapy protocol. The limitation includes considering only healthy subjects and using separate systems for modules. Future work will focus on including elders and sports or gym professionals who represent a significant demographic target and the use of one dedicated module to avoid calibration and timing errors.

## Figures and Tables

**Table 1:** Overview of relevant studies, highlighting machine learning approaches on knee performance assessment and associated conditions of classification (RF: Random Forest, MLP: Multilayer Perceptron, LDA: Linear Discriminant Analysis, SVM: Support Vector Machine, LMBP: Levenberg–Marquardt Backpropagation, CNN: Convolutional Neural Network, MARS: Multivariate Adaptive Regression Splines, DT: Decision Tree, KNN: K-Nearest Neighbors, ET: Extra Tree, DHAT-LSTM: Dual Head Attentional Transformer-Long Short Term Memory, LSTM-ADAM: LSTM-Adaptive Moment Estimation, LSTM-SGD: LSTM-Stochastic Gradient Descent, KNN-DTW: KNN-Dynamic Time Warping, RBF: Radial Basis Function, ELA: Ensemble Learning-based Adaboost, NB: Naive Bayes).

Study	Features	Methods	Target Conditions	Accuracy
Zapata et al. [15]	Lower limb joint angles	MLP/SVM	Squat posture recognition	MLP: 92% SVM: 72%
Liu et al. [16]	Knee joint angles	SVM	Knee joint injury assessment	Not reported
Girase et al. [17]	Lower limb joint angles	SVM/RF/MLP	Assessment of lower limb pathologies	MLP: 73%
Zeng et al. [5]	6-DOF-based knee joint angles	SVM/RBF	Knee gait pattern classification	2-fold RBF: 95.7% 1-fold RBF: 97.9%
Taborri et al. [18]	Lower limb joint angles	SVM/KNN/DT	ACL injury assessment	SVM: 96% KNN: 67% DT: Not reported
Zeng et al. [19]	Lower limb joint angles	SVM/DT/KNN/NB/ELA	Gait classification including patients with ACL-deficient and intact knees	SVM: (91~96)% DT: (77~83)% KNN: (78~88)% NB: (57~61)% ELA: (73~79)%
Vijayvargiya et al. [20]	EMG signals	SVM/DT/KNN/RF/ET	Knee abnormality classification	SVM: 70.1% DT: 70% KNN: 79.3% RF: 88.8% ET: 91.3%
Liu et al. [21]	EMG, acceleration, knee angles, foot pressure	LDA/SVM/LM-BP	Lower limb movement intention recognition	LDA: 92.46%
Moghadam et al. [22]	Kinematic/kinetic joint data, GRF, muscle forces	CNN/RF/SVM/MARS	ML model comparison for locomotion prediction	Not reported
Oh et al. [23]	Shoulder, knee and ankle joint angles and CoP data	SVM/NB	Squat posture recognition	SVM: 95.61% NB: 81.82%
Vijayvargiya et al. [24]	EMG signals	Conv-LSTM	Automated knee abnormality detection	LSTM: 98.61%
Narayan et al. [25]	knee joints and EMG	LSTM-SGD/LSTM-ADAM	Healthy and impaired gait assessment	LSTM-SGD: 79.18% LSTM-ADAM: 91.72%
Mohsen et al. [26]	Upper and lower limb joints	DHAT-LSTM	Gait dysfunction classification	DHAT-LSTM: 81%
Felix et al. [27]	Upper and lower limb joints	SVM/KNN-DTW/Conv-LSTM	Movement error classification	Squat-SVM: 56% Squat-KNN-DTW: 80% Squat-Conv-LSTM: 80%
Junhui at al. [28]	Upper and lower limb joints	RF/Conv-LSTM	Gait and squat assessment	RF: 91.3% Conv-LSTM: 95.18%

**Table 2:** Performance comparison of different feature sets using correlation technique.

Feature Sets	AUC	Recall	F1 Score	Precision	Accuracy (%)
1,2,3,4,5	0.92	0.75	0.80	0.88	96.04
1,2,3	0.91	0.75	0.79	0.90	96.13
1,3,5	0.92	0.47	0.48	0.47	93.40
4,5	0.64	0.50	0.48	0.47	93.57
2,4	0.77	0.50	0.49	0.57	93.60

**Table 3:** Performance comparison of different feature sets using the SVM technique.

Feature Sets	AUC	Recall	F1 Score	Precision	Accuracy (%)
1,2,3,4,5	0.94	0.46	0.63	1	96.54
1,2,3	0.94	0.46	0.63	1	96.51
1,3,5	0.94	0.47	0.64	1	96.59
4,5	0.77	0.46	0.63	1	96.54
2,4	0.83	0.46	0.63	1	96.51

## Data Availability

The original contributions presented in this study are included in the article/Supplementary Material. Further inquiries can be directed to the corresponding author.

## Supplementary Materials

The following supporting information can be downloaded at: https://www.mdpi.com/article/10.3390/s25196074/s1, S1: The file includes figures showing the AUC, confusion matrix and five-fold cross-validation of the LSTM network; S2: The file includes the confusion matrix figures of a single-fold evaluation of the SVM network.

## Abbreviations

The following abbreviations are used in this manuscript:

LSTM	Long Short-Term Memory
SVM	Support Vector Machine
ACL	Anterior Cruciate Ligament
PCL	Posterior Cruciate Ligament
KI	Knee Instability
NKI	Non-Knee Instability
PGM	Pneumatic Gel Muscle
VR	Virtual Reality
ANN	Artificial Neural Network
RNN	Recurrent Neural Network
IMU	Inertial Measurement Unit
CoP	Center of Pressure
FPS	Frames Per Second
KS	Knee Shakiness
KD	Knee Distance
SD	Squat Depth
SV	Sway Velocity
SA	Sway Area
RBF	Radial Basis Function
SMO	Sequential Minimal Optimization
