# Supplementary figures and images for: A Preliminary Data-Driven Approach for Classifying Knee Instability During Subject-Specific Exercise-Based Game with Squat Motions
Source: Sensors (Basel). 2025 Oct 2;25(19):6074. doi: 10.3390/s25196074 (PMC12526614; doi:10.3390/s25196074)

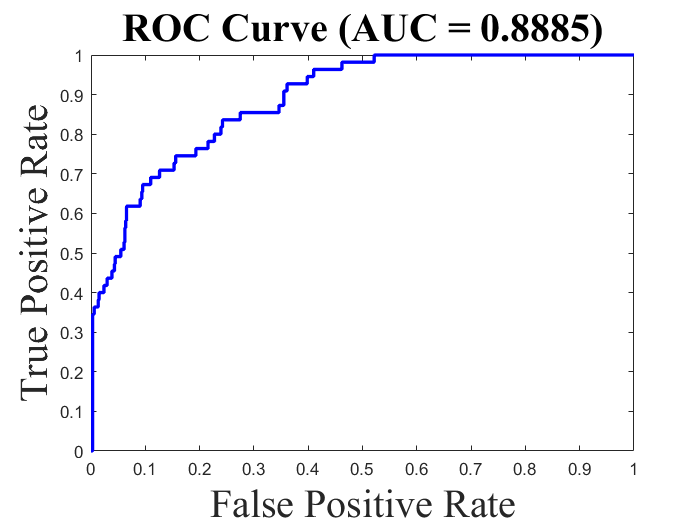

Supplement: Supplementary file 1 [file sensors-25-06074-s001.zip › Supplementary_files/S1/lstm_123/AUC/KW_fdepthcamera123_roc1.png]

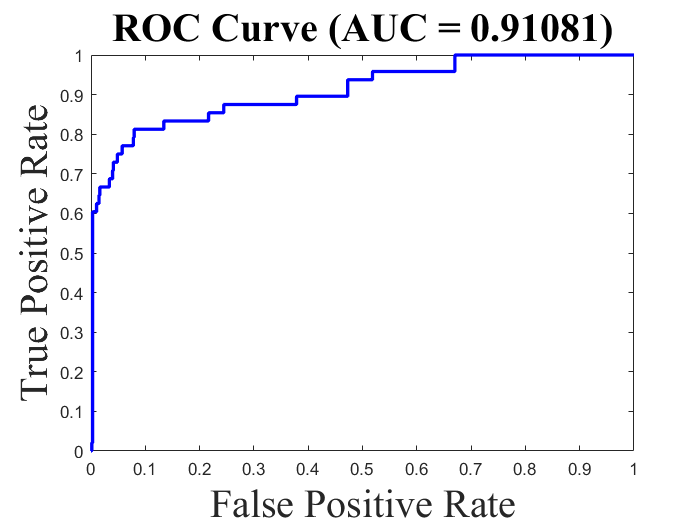

Supplement: Supplementary file 1 [file sensors-25-06074-s001.zip › Supplementary_files/S1/lstm_123/AUC/KW_fdepthcamera123_roc2.png]

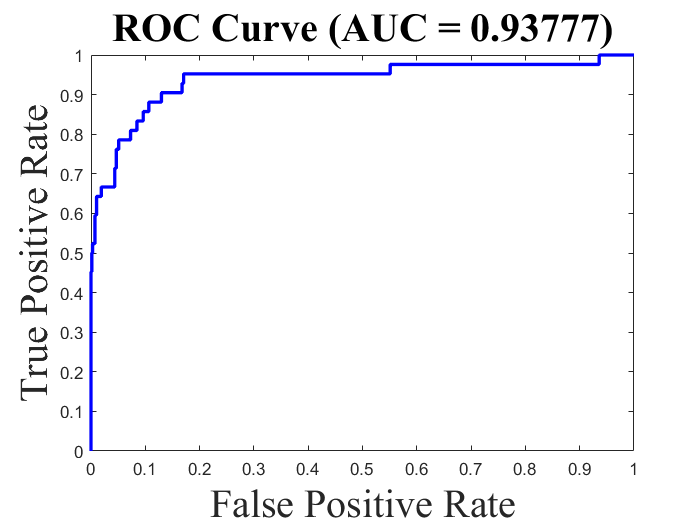

Supplement: Supplementary file 1 [file sensors-25-06074-s001.zip › Supplementary_files/S1/lstm_123/AUC/KW_fdepthcamera123_roc3.png]

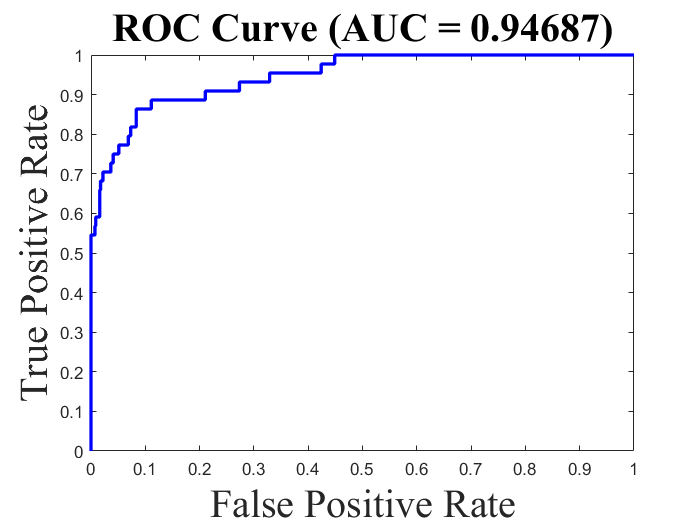

Supplement: Supplementary file 1 [file sensors-25-06074-s001.zip › Supplementary_files/S1/lstm_123/AUC/KW_fdepthcamera123_roc4.png]

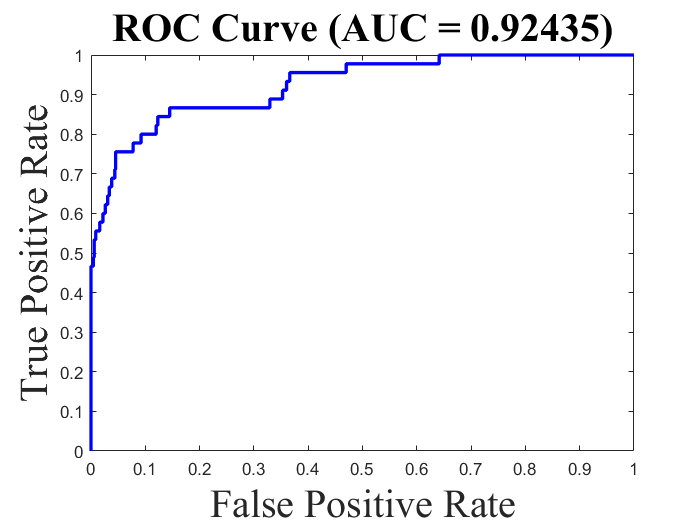

Supplement: Supplementary file 1 [file sensors-25-06074-s001.zip › Supplementary_files/S1/lstm_123/AUC/KW_fdepthcamera123_roc5.png]

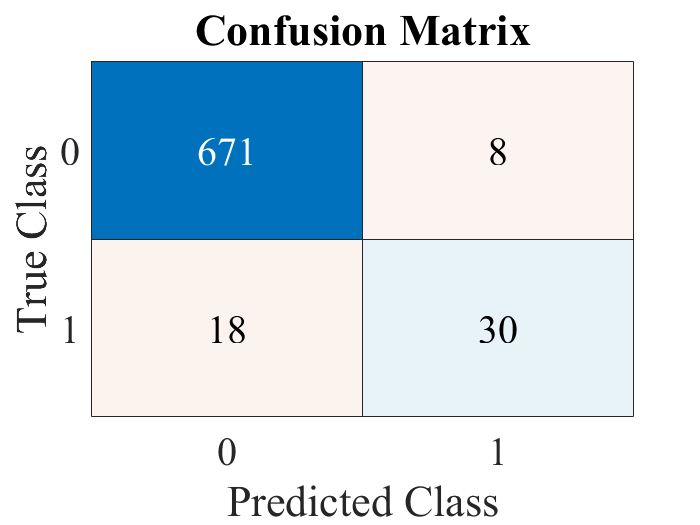

Supplement: Supplementary file 1 [file sensors-25-06074-s001.zip › Supplementary_files/S1/lstm_123/Confusion_Matrix/KW_fdepthcamera123_cm1.png]

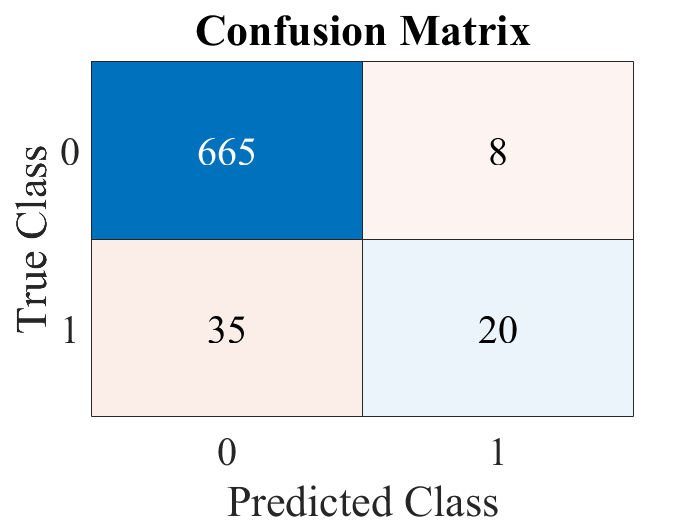

Supplement: Supplementary file 1 [file sensors-25-06074-s001.zip › Supplementary_files/S1/lstm_123/Confusion_Matrix/KW_fdepthcamera123_cm2.png]

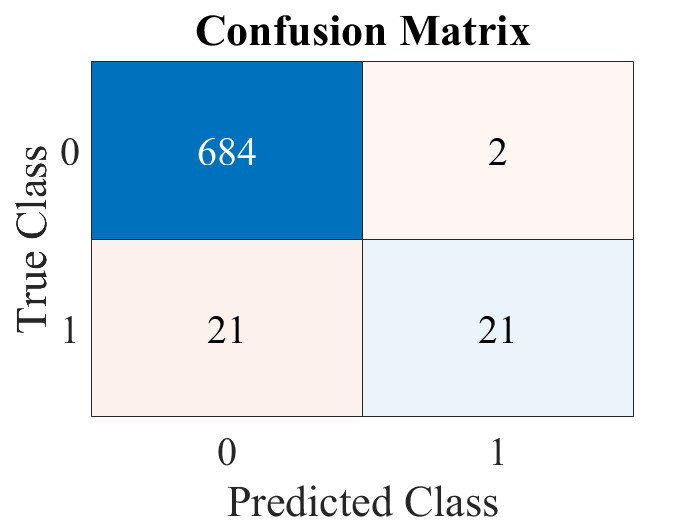

Supplement: Supplementary file 1 [file sensors-25-06074-s001.zip › Supplementary_files/S1/lstm_123/Confusion_Matrix/KW_fdepthcamera123_cm3.png]

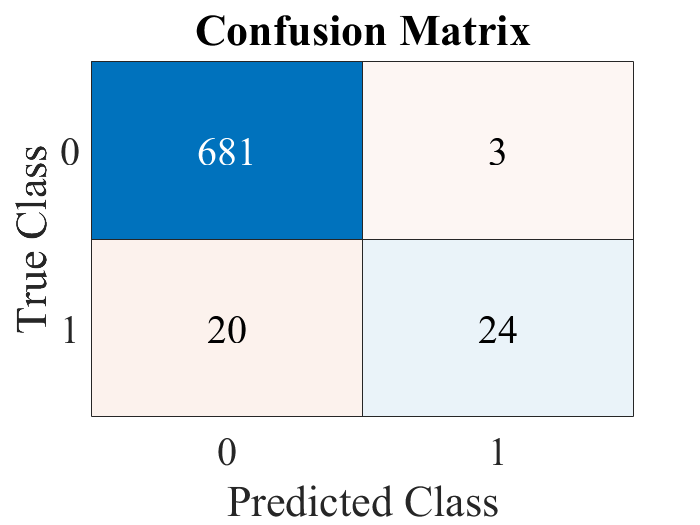

Supplement: Supplementary file 1 [file sensors-25-06074-s001.zip › Supplementary_files/S1/lstm_123/Confusion_Matrix/KW_fdepthcamera123_cm4.png]

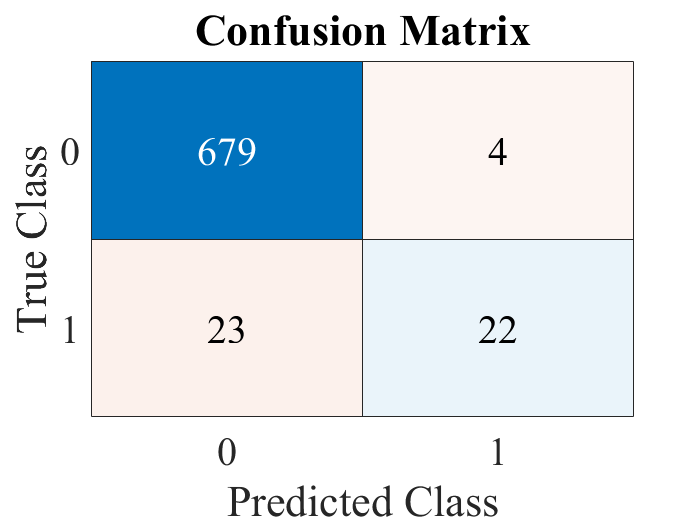

Supplement: Supplementary file 1 [file sensors-25-06074-s001.zip › Supplementary_files/S1/lstm_123/Confusion_Matrix/KW_fdepthcamera123_cm5.png]

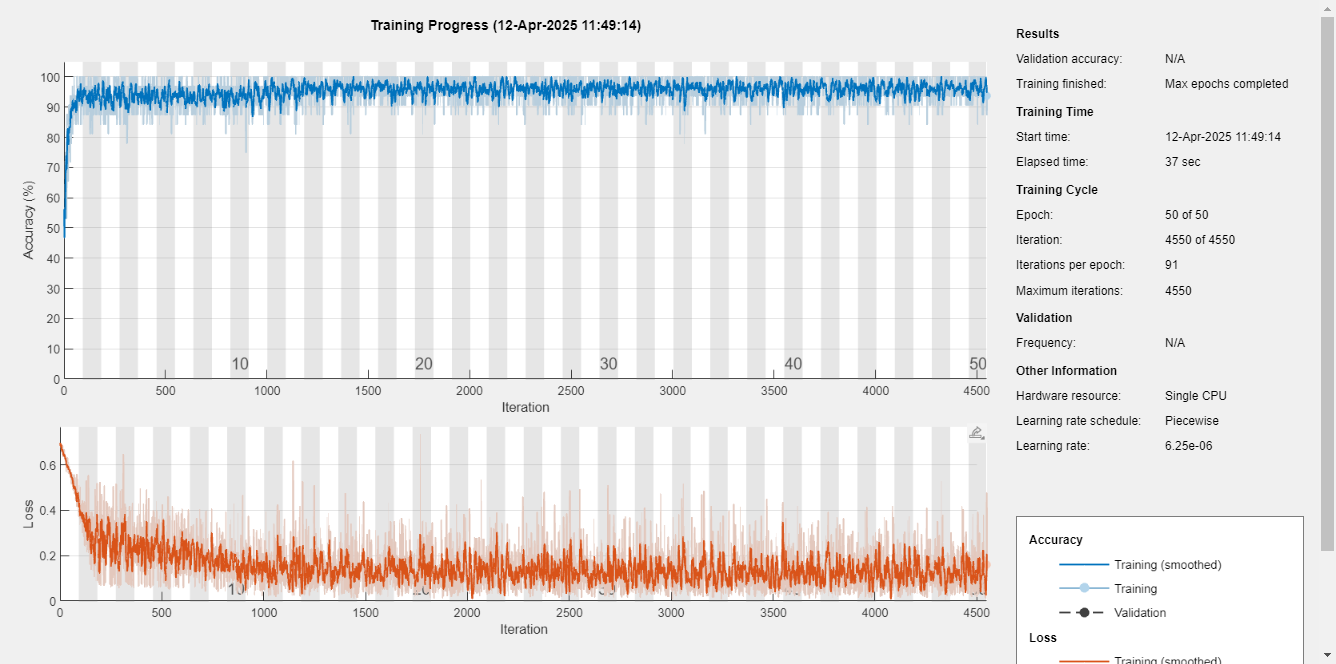

Supplement: Supplementary file 1 [file sensors-25-06074-s001.zip › Supplementary_files/S1/lstm_123/Network_Cross_Validation/KW_fdepthcamera123_ncv1.png]

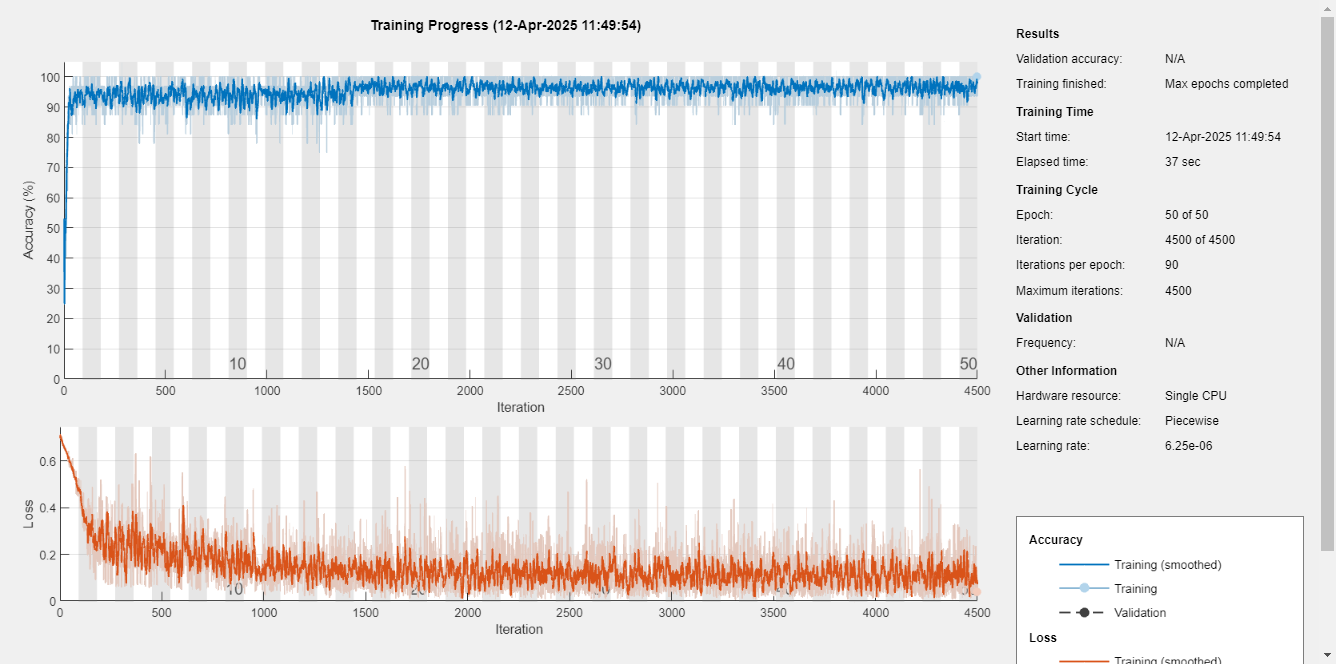

Supplement: Supplementary file 1 [file sensors-25-06074-s001.zip › Supplementary_files/S1/lstm_123/Network_Cross_Validation/KW_fdepthcamera123_ncv2.png]

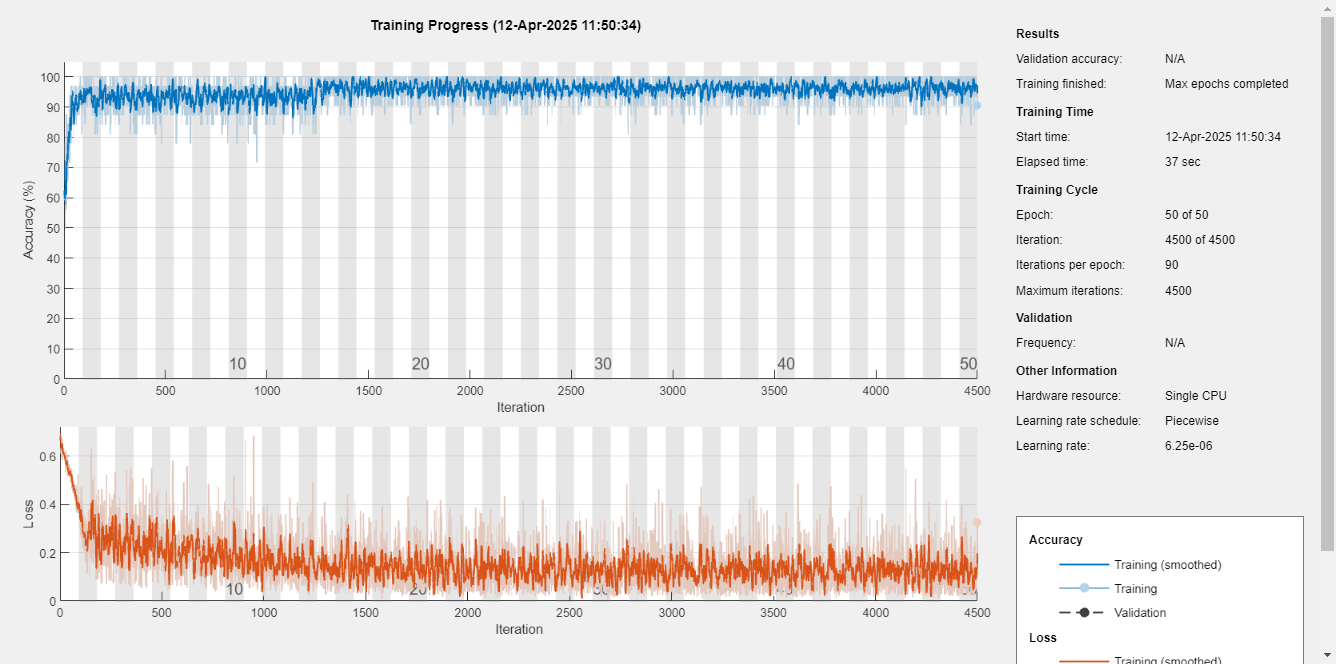

Supplement: Supplementary file 1 [file sensors-25-06074-s001.zip › Supplementary_files/S1/lstm_123/Network_Cross_Validation/KW_fdepthcamera123_ncv3.png]

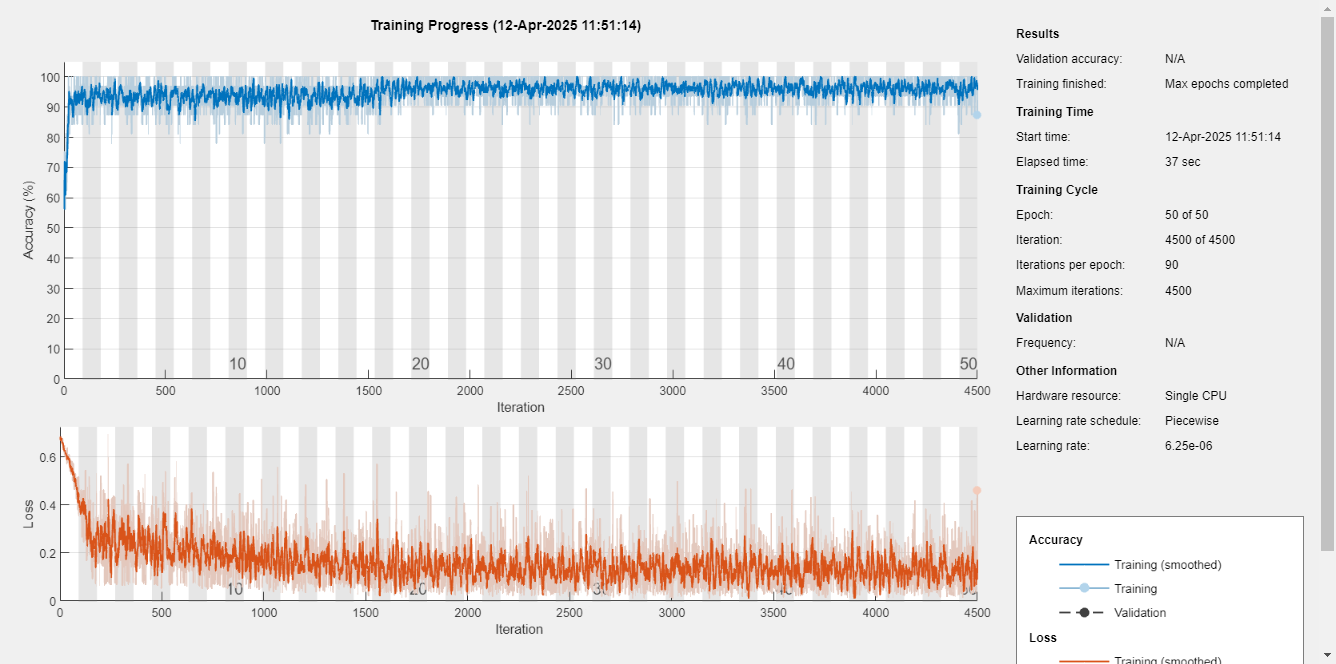

Supplement: Supplementary file 1 [file sensors-25-06074-s001.zip › Supplementary_files/S1/lstm_123/Network_Cross_Validation/KW_fdepthcamera123_ncv4.png]

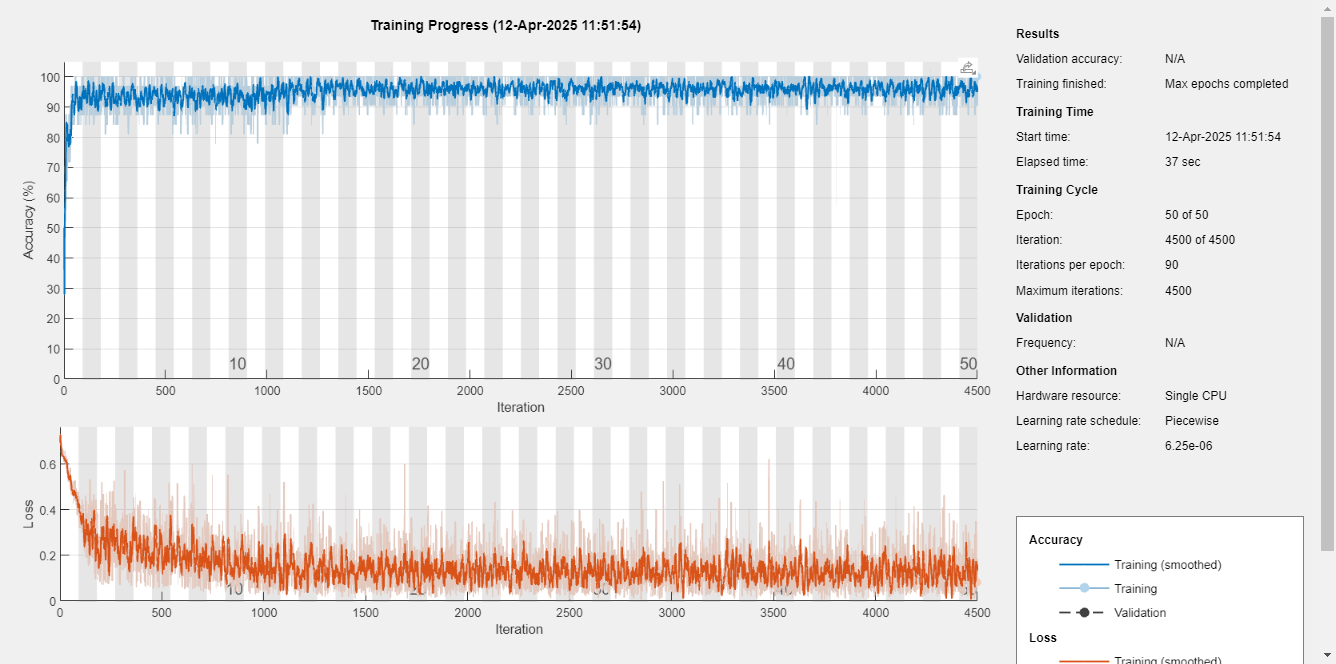

Supplement: Supplementary file 1 [file sensors-25-06074-s001.zip › Supplementary_files/S1/lstm_123/Network_Cross_Validation/KW_fdepthcamera123_ncv5.png]

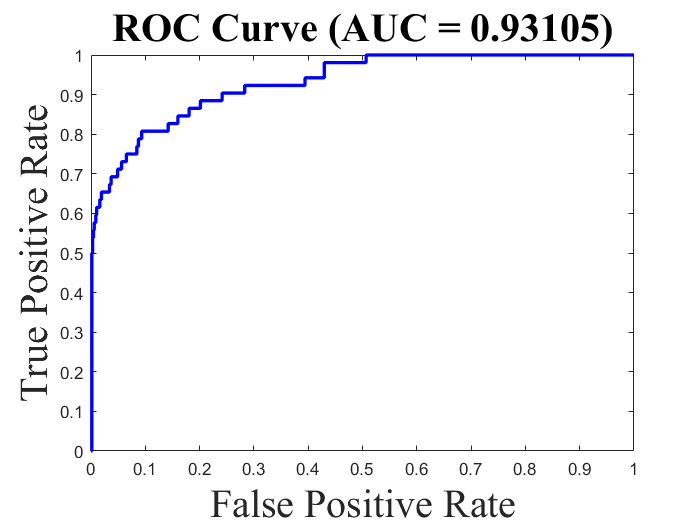

Supplement: Supplementary file 1 [file sensors-25-06074-s001.zip › Supplementary_files/S1/lstm_12345/AUC/KW_fcopanddepthcamera12345_roc1.png]

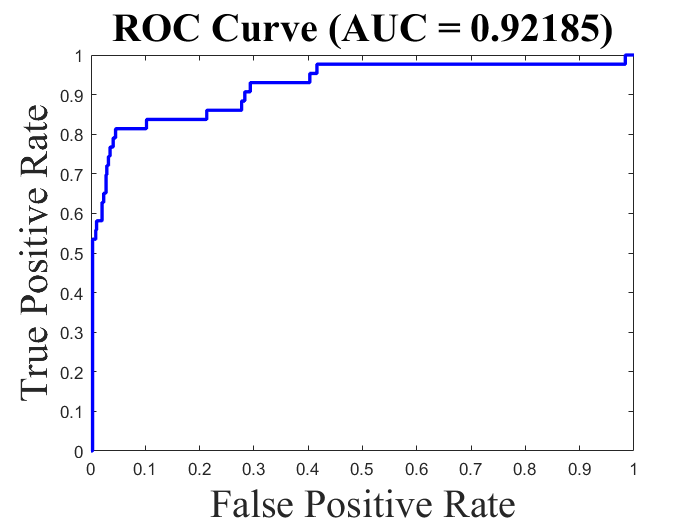

Supplement: Supplementary file 1 [file sensors-25-06074-s001.zip › Supplementary_files/S1/lstm_12345/AUC/KW_fcopanddepthcamera12345_roc2.png]

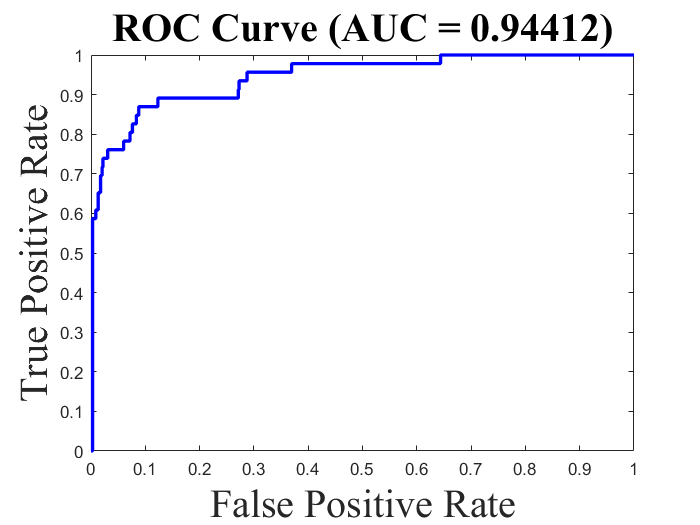

Supplement: Supplementary file 1 [file sensors-25-06074-s001.zip › Supplementary_files/S1/lstm_12345/AUC/KW_fcopanddepthcamera12345_roc3.png]

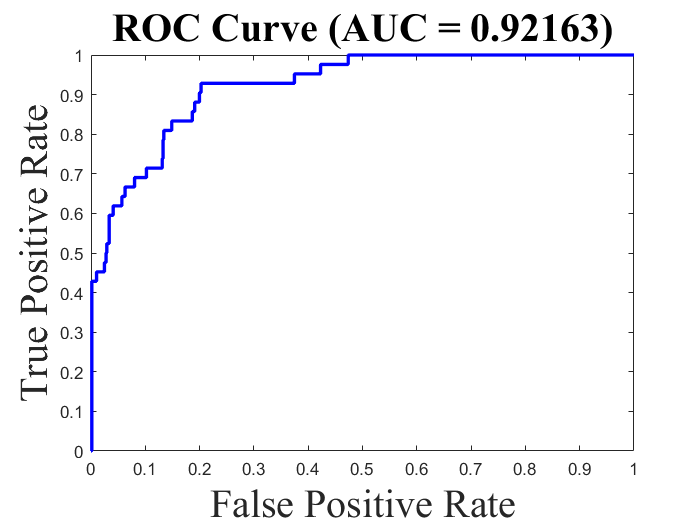

Supplement: Supplementary file 1 [file sensors-25-06074-s001.zip › Supplementary_files/S1/lstm_12345/AUC/KW_fcopanddepthcamera12345_roc4.png]

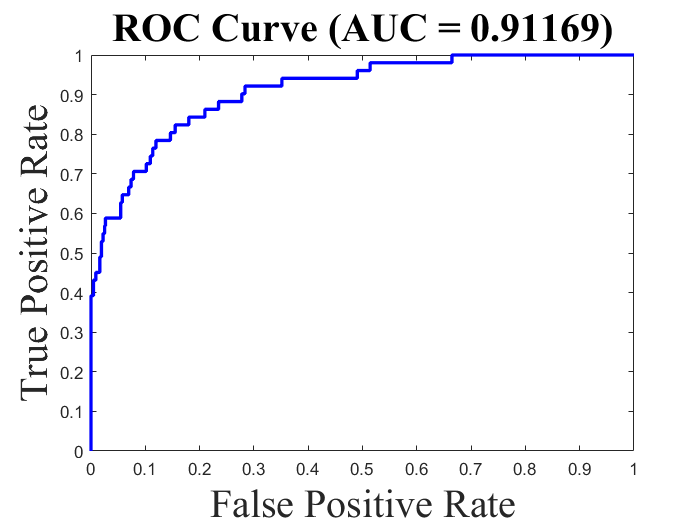

Supplement: Supplementary file 1 [file sensors-25-06074-s001.zip › Supplementary_files/S1/lstm_12345/AUC/KW_fcopanddepthcamera12345_roc5.png]

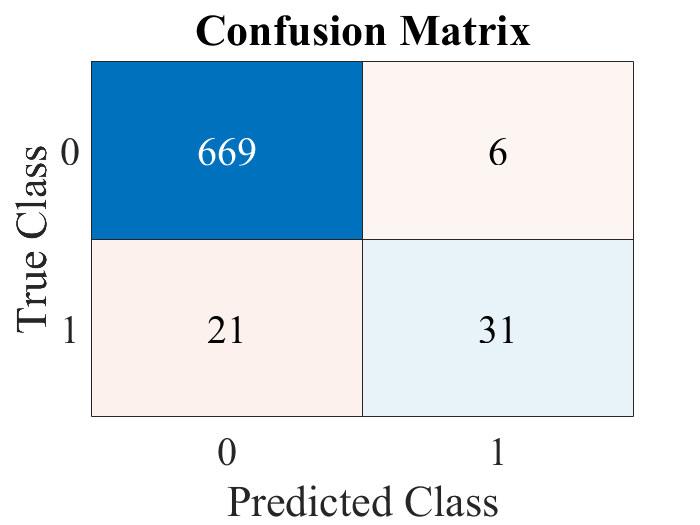

Supplement: Supplementary file 1 [file sensors-25-06074-s001.zip › Supplementary_files/S1/lstm_12345/Confusion_Matrix/KW_fcopanddepthcamera12345_cm1.png]

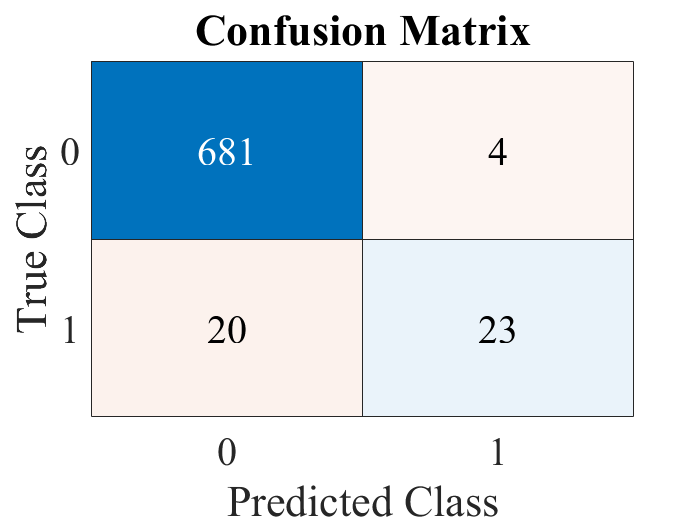

Supplement: Supplementary file 1 [file sensors-25-06074-s001.zip › Supplementary_files/S1/lstm_12345/Confusion_Matrix/KW_fcopanddepthcamera12345_cm2.png]

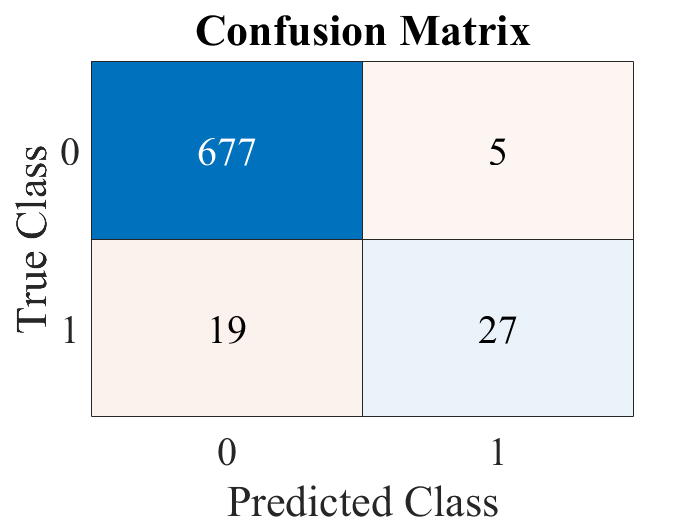

Supplement: Supplementary file 1 [file sensors-25-06074-s001.zip › Supplementary_files/S1/lstm_12345/Confusion_Matrix/KW_fcopanddepthcamera12345_cm3.png]

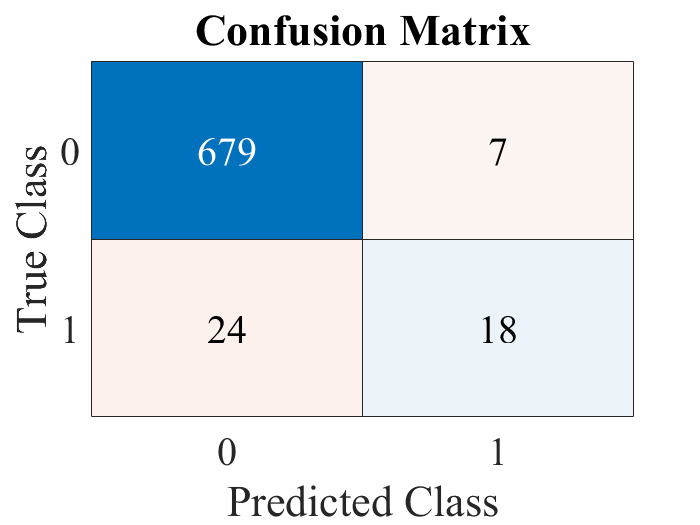

Supplement: Supplementary file 1 [file sensors-25-06074-s001.zip › Supplementary_files/S1/lstm_12345/Confusion_Matrix/KW_fcopanddepthcamera12345_cm4.png]

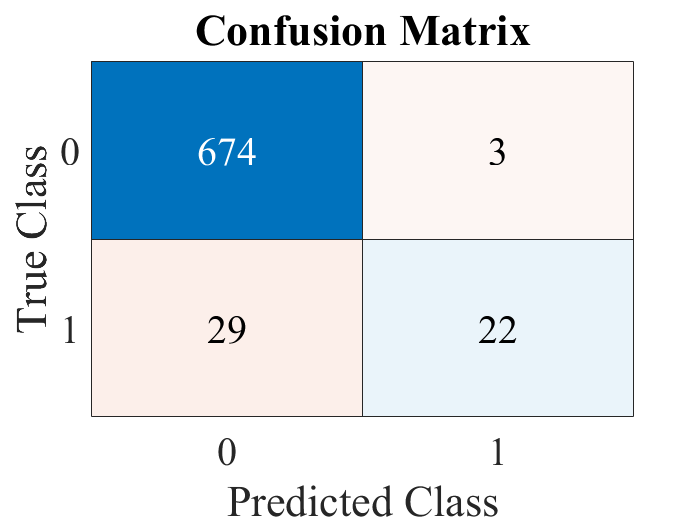

Supplement: Supplementary file 1 [file sensors-25-06074-s001.zip › Supplementary_files/S1/lstm_12345/Confusion_Matrix/KW_fcopanddepthcamera12345_cm5.png]

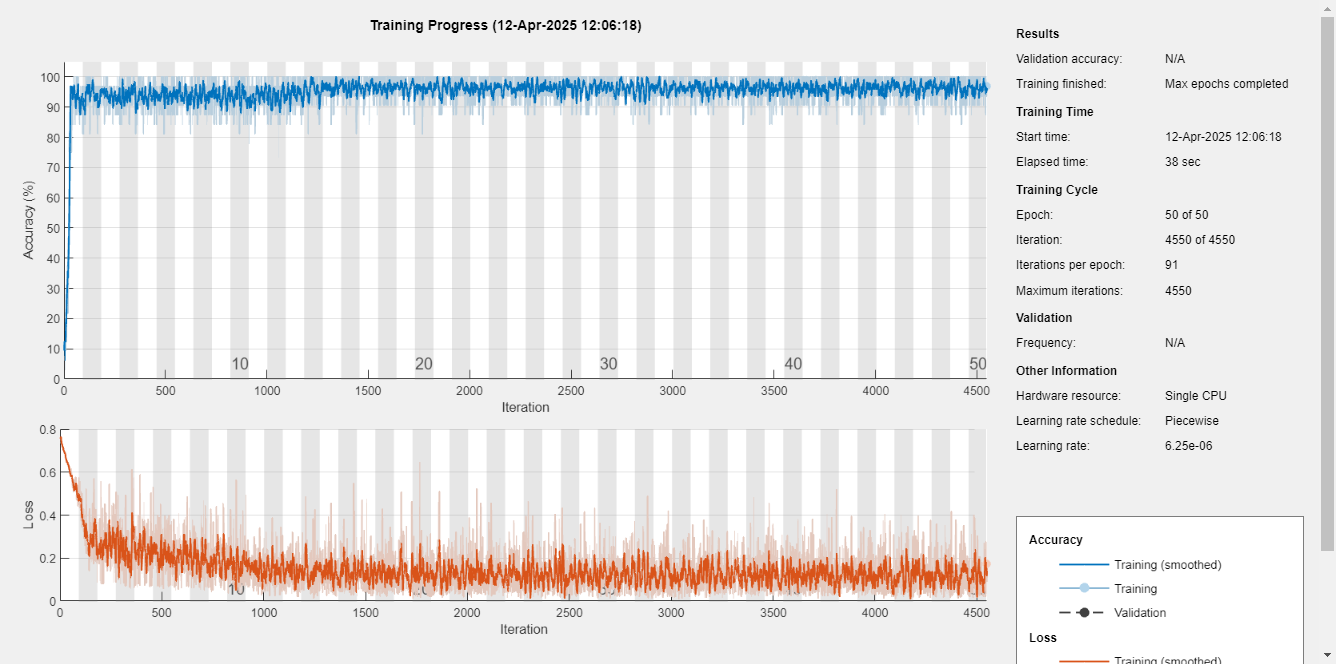

Supplement: Supplementary file 1 [file sensors-25-06074-s001.zip › Supplementary_files/S1/lstm_12345/Network_Cross_Validation/KW_fcopanddepthcamera12345_ncv1.png]

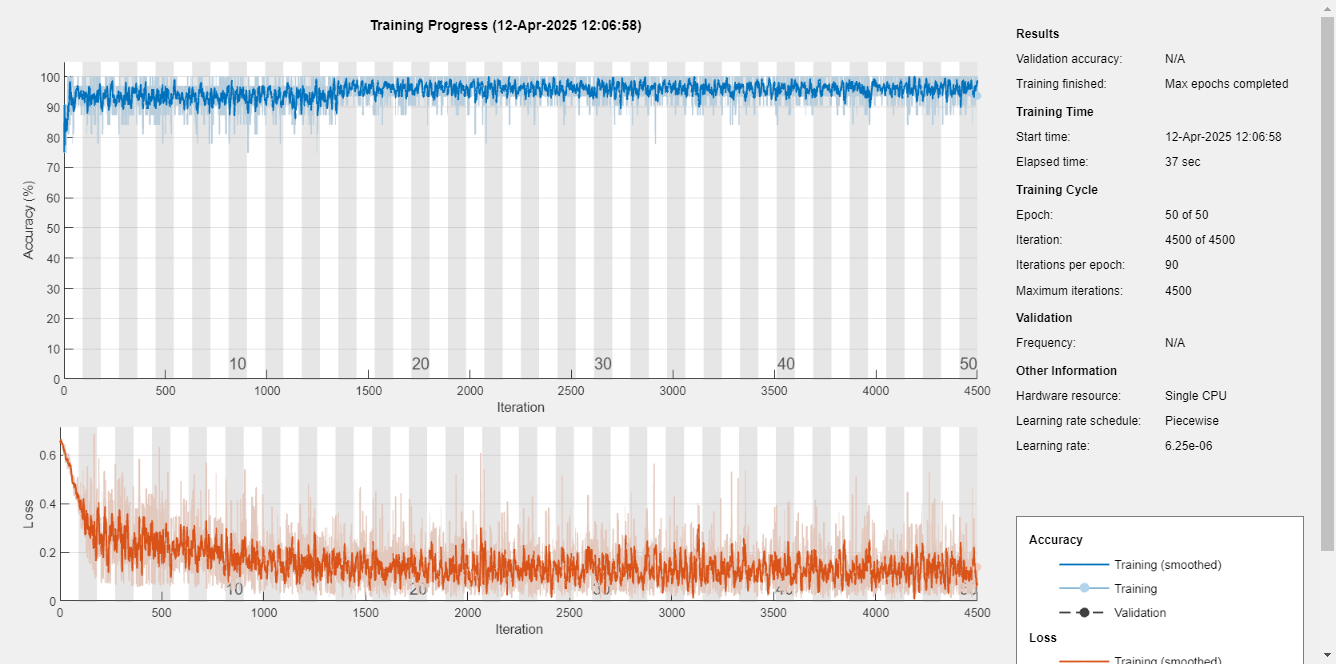

Supplement: Supplementary file 1 [file sensors-25-06074-s001.zip › Supplementary_files/S1/lstm_12345/Network_Cross_Validation/KW_fcopanddepthcamera12345_ncv2.png]

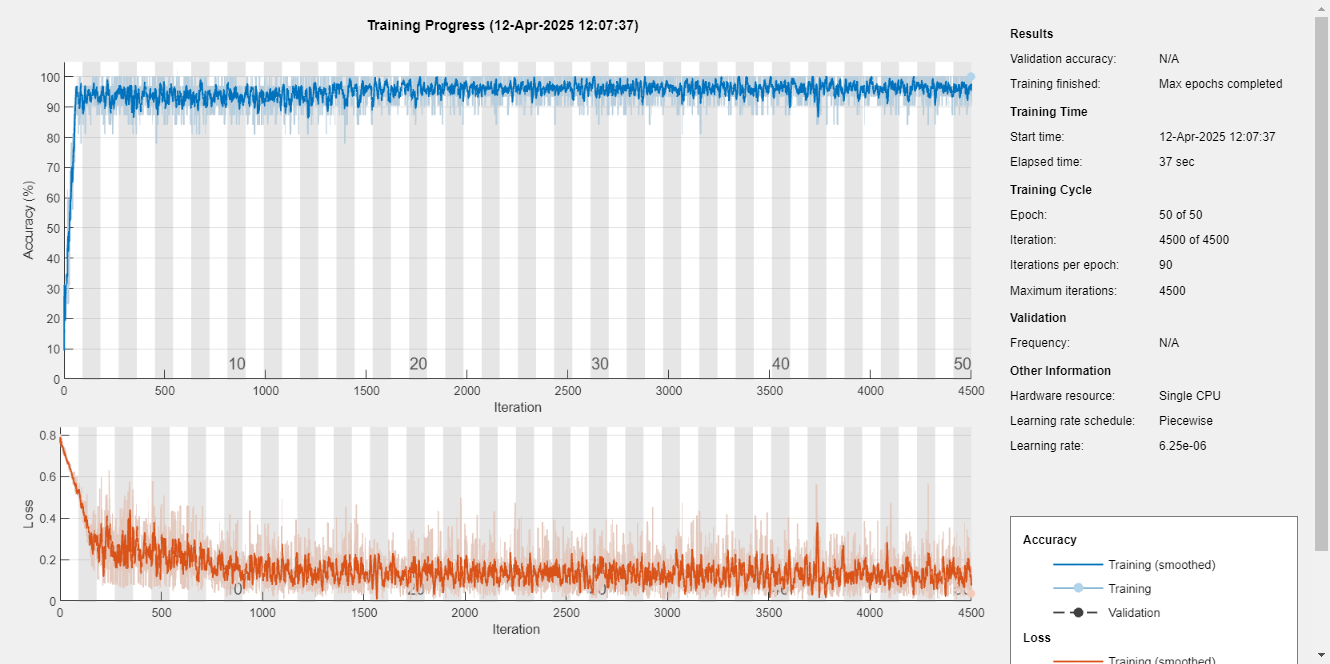

Supplement: Supplementary file 1 [file sensors-25-06074-s001.zip › Supplementary_files/S1/lstm_12345/Network_Cross_Validation/KW_fcopanddepthcamera12345_ncv3.png]

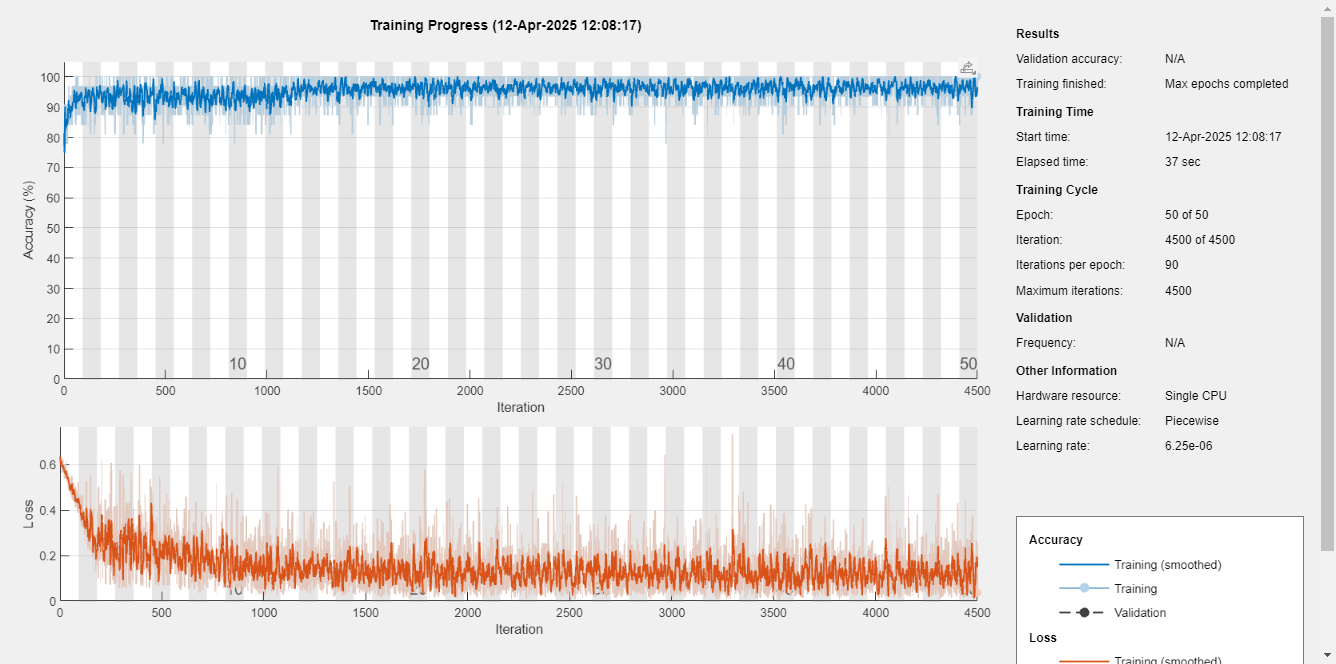

Supplement: Supplementary file 1 [file sensors-25-06074-s001.zip › Supplementary_files/S1/lstm_12345/Network_Cross_Validation/KW_fcopanddepthcamera12345_ncv4.png]

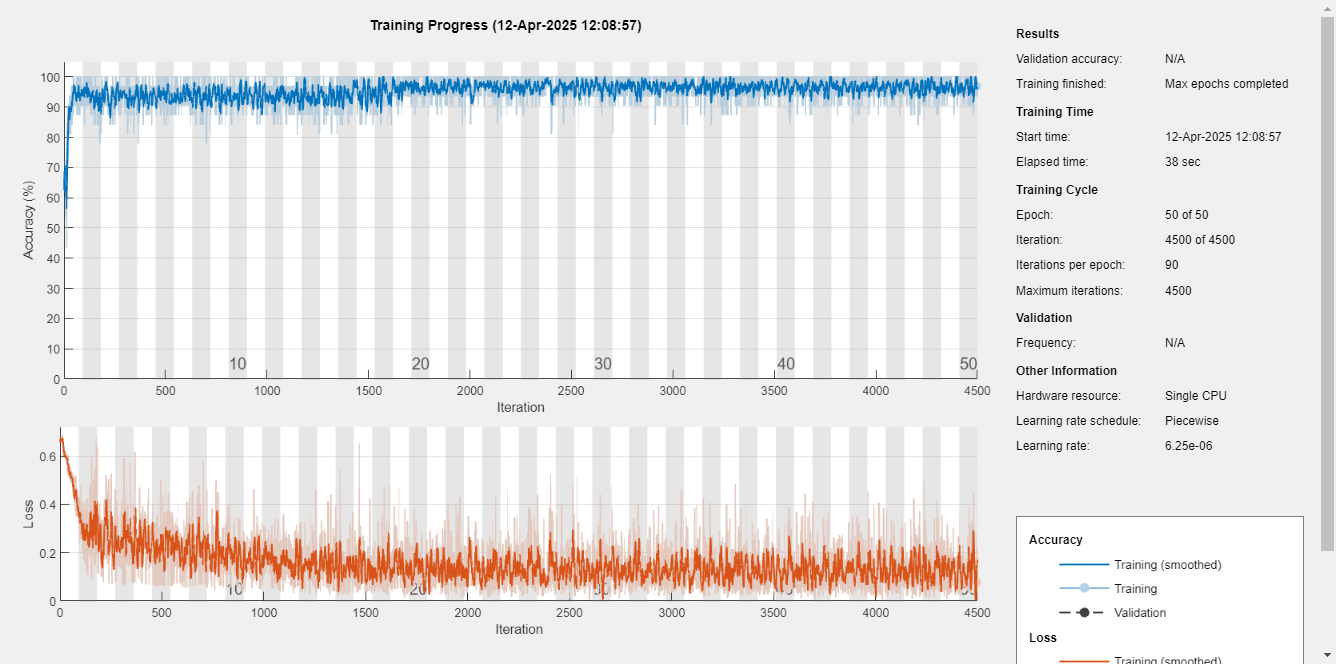

Supplement: Supplementary file 1 [file sensors-25-06074-s001.zip › Supplementary_files/S1/lstm_12345/Network_Cross_Validation/KW_fcopanddepthcamera12345_ncv5.png]

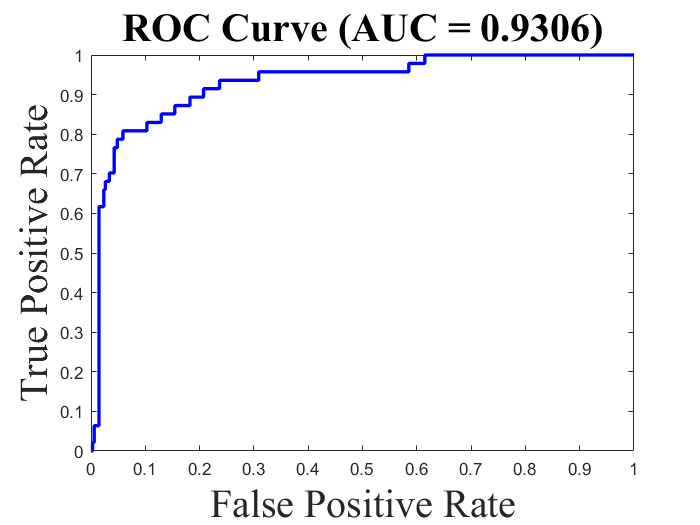

Supplement: Supplementary file 1 [file sensors-25-06074-s001.zip › Supplementary_files/S1/lstm_135/AUC/KW_frho135_roc1.png]

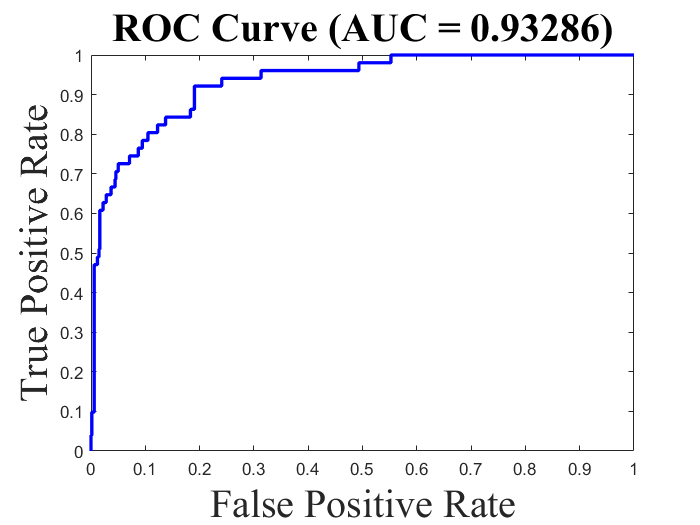

Supplement: Supplementary file 1 [file sensors-25-06074-s001.zip › Supplementary_files/S1/lstm_135/AUC/KW_frho135_roc2.png]

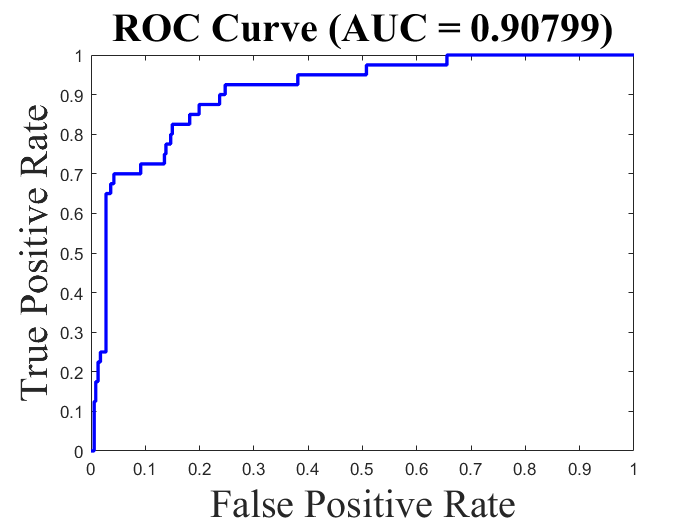

Supplement: Supplementary file 1 [file sensors-25-06074-s001.zip › Supplementary_files/S1/lstm_135/AUC/KW_frho135_roc3.png]

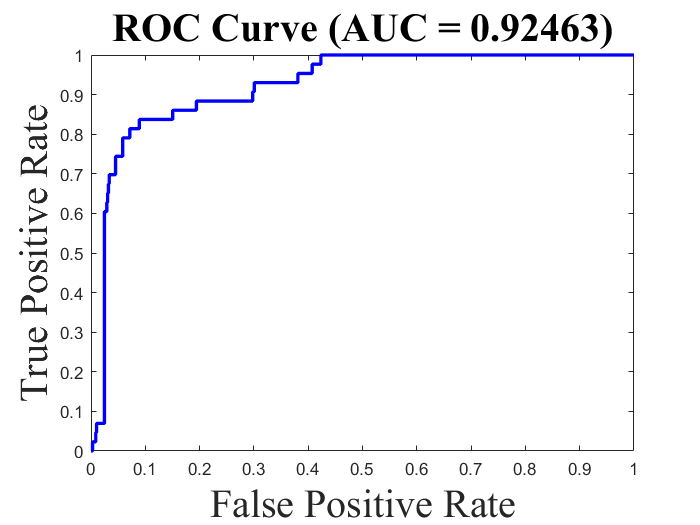

Supplement: Supplementary file 1 [file sensors-25-06074-s001.zip › Supplementary_files/S1/lstm_135/AUC/KW_frho135_roc4.png]

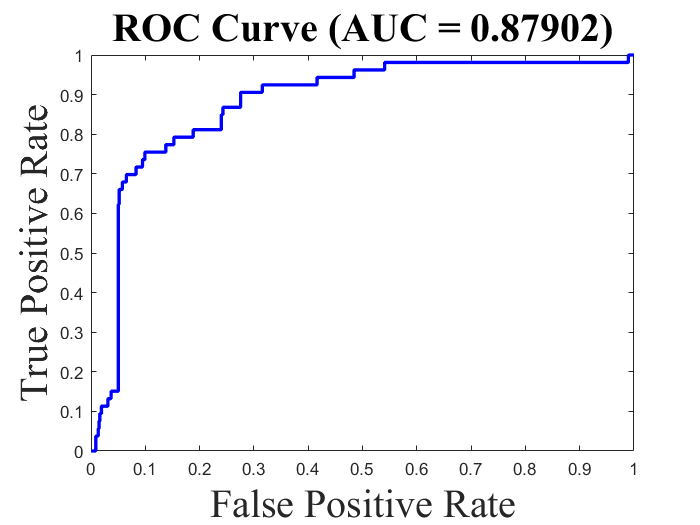

Supplement: Supplementary file 1 [file sensors-25-06074-s001.zip › Supplementary_files/S1/lstm_135/AUC/KW_frho135_roc5.png]

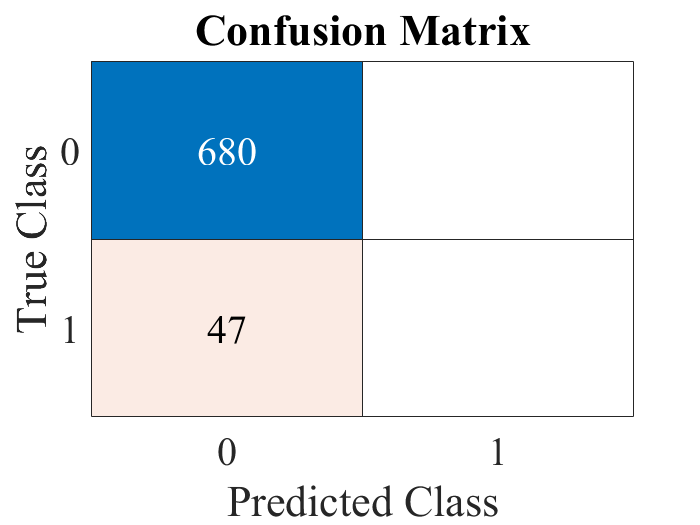

Supplement: Supplementary file 1 [file sensors-25-06074-s001.zip › Supplementary_files/S1/lstm_135/Confusion_Matrix/KW_frho135_cm1.png]

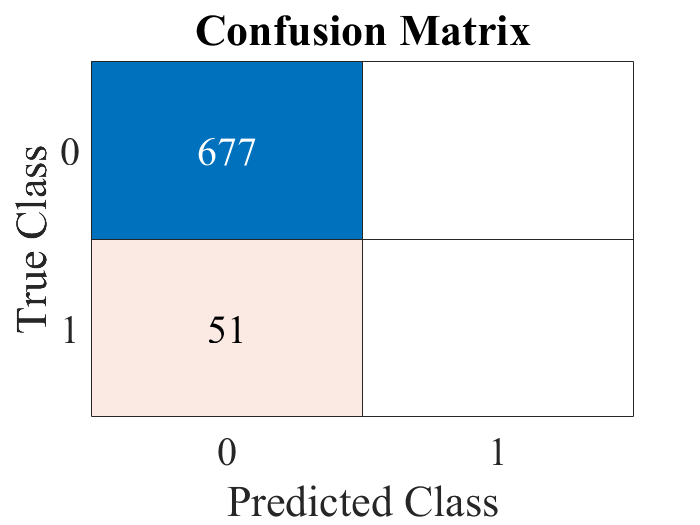

Supplement: Supplementary file 1 [file sensors-25-06074-s001.zip › Supplementary_files/S1/lstm_135/Confusion_Matrix/KW_frho135_cm2.png]

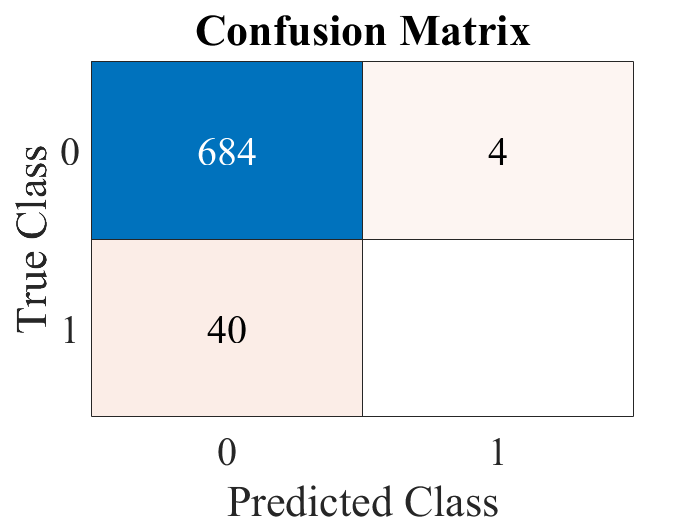

Supplement: Supplementary file 1 [file sensors-25-06074-s001.zip › Supplementary_files/S1/lstm_135/Confusion_Matrix/KW_frho135_cm3.png]

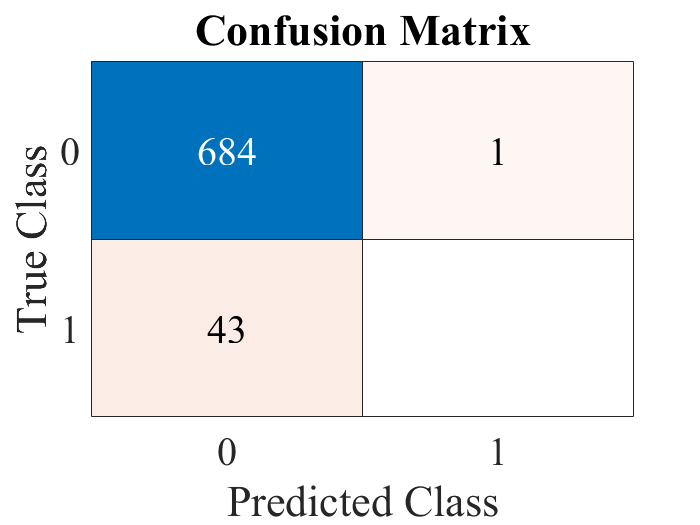

Supplement: Supplementary file 1 [file sensors-25-06074-s001.zip › Supplementary_files/S1/lstm_135/Confusion_Matrix/KW_frho135_cm4.png]

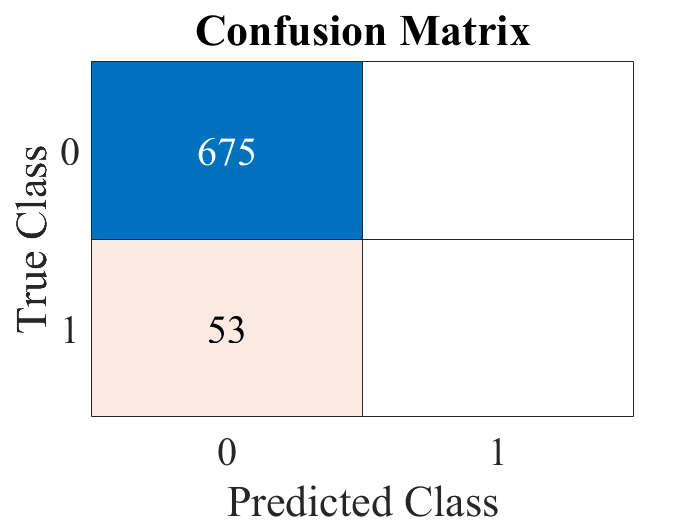

Supplement: Supplementary file 1 [file sensors-25-06074-s001.zip › Supplementary_files/S1/lstm_135/Confusion_Matrix/KW_frho135_cm5.png]

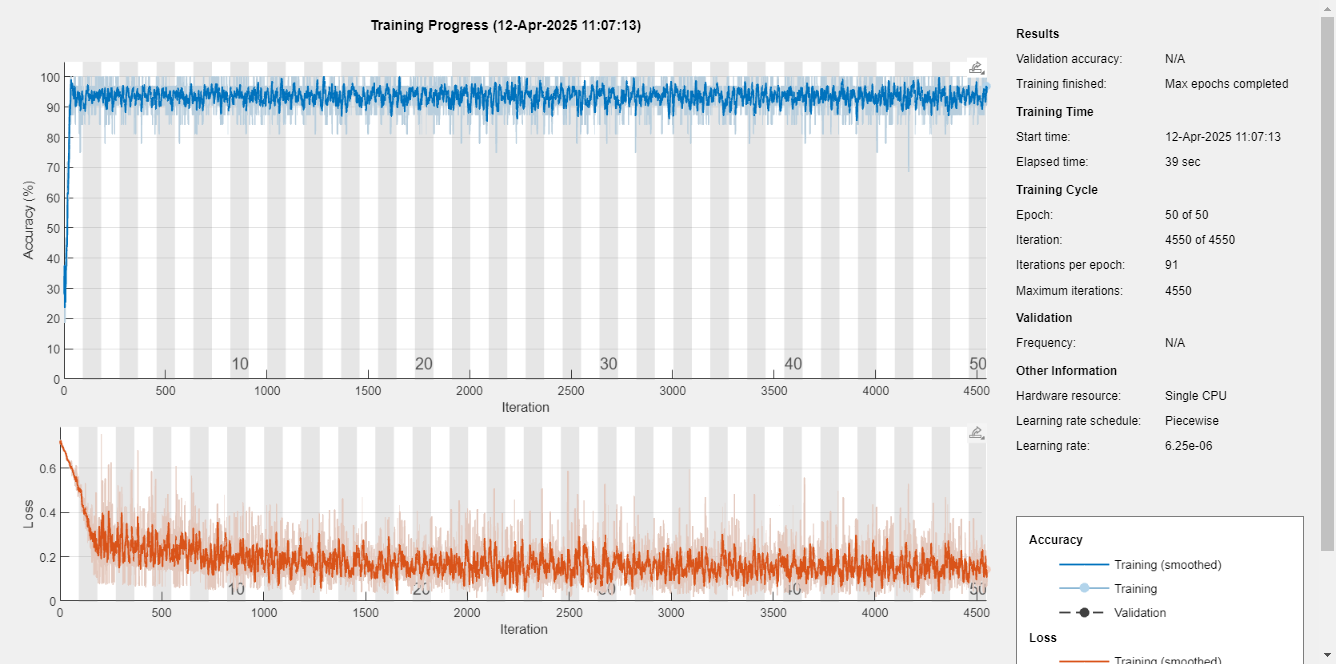

Supplement: Supplementary file 1 [file sensors-25-06074-s001.zip › Supplementary_files/S1/lstm_135/Network_Cross_Validation/KW_frho135_ncv1.png]

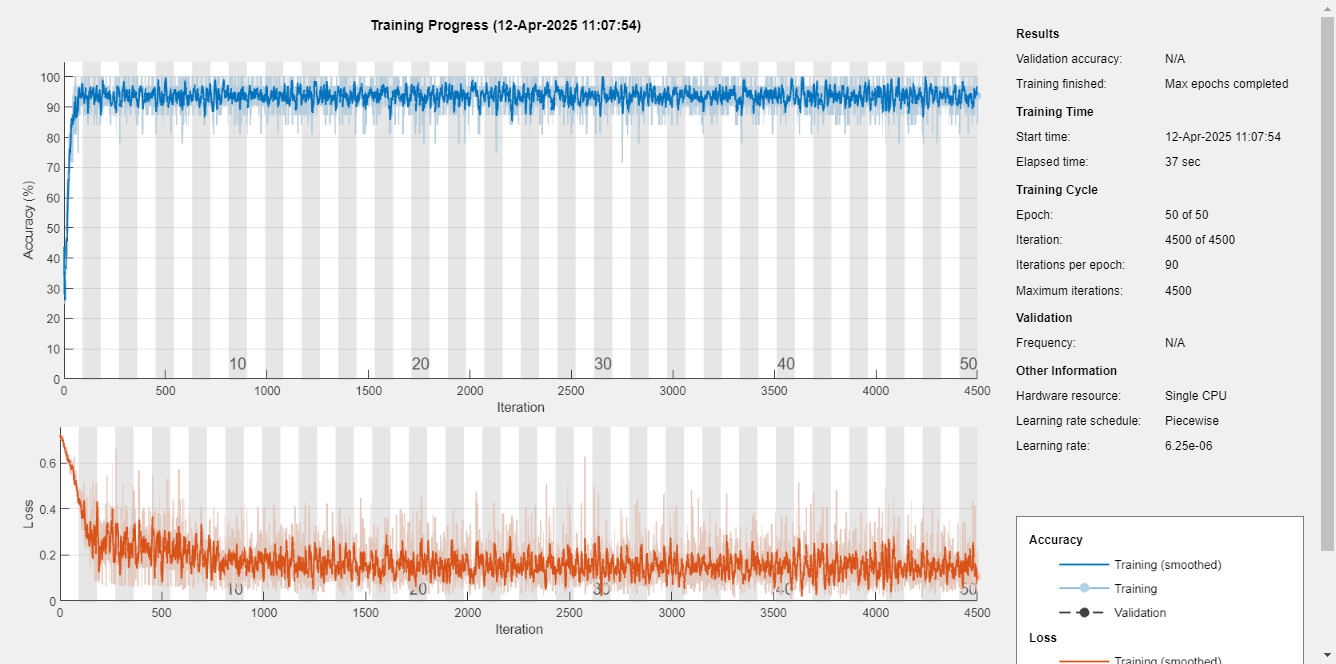

Supplement: Supplementary file 1 [file sensors-25-06074-s001.zip › Supplementary_files/S1/lstm_135/Network_Cross_Validation/KW_frho135_ncv2.png]

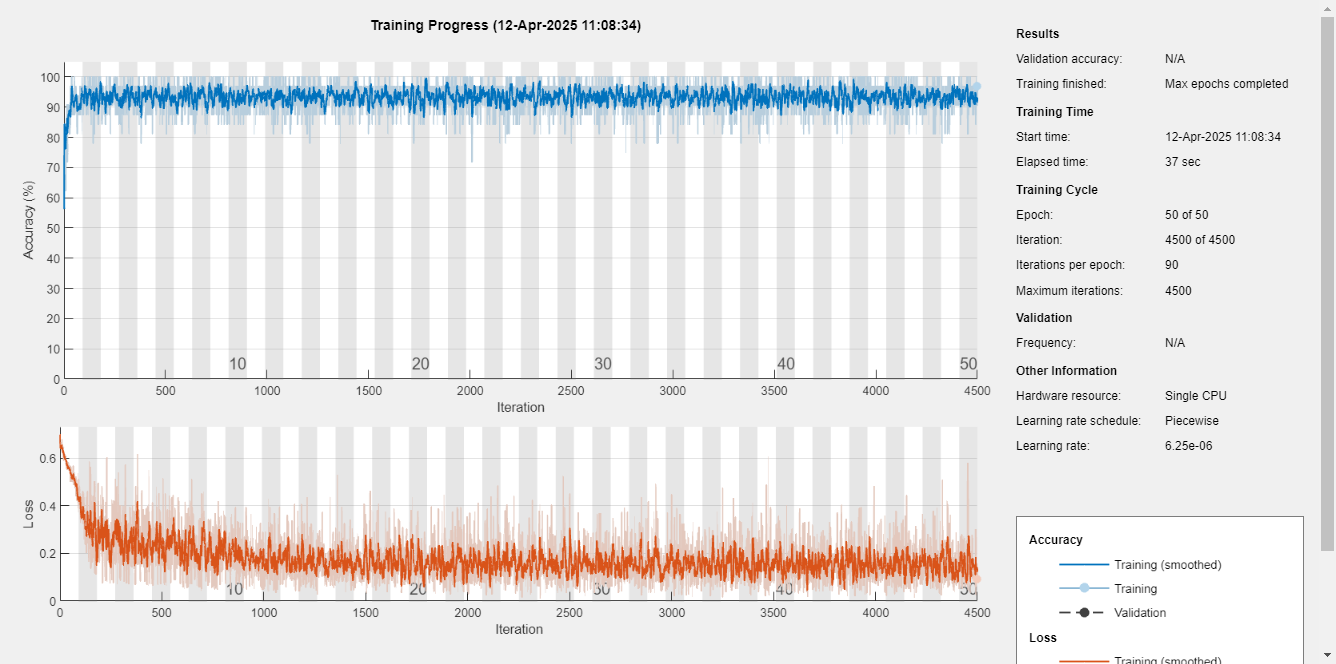

Supplement: Supplementary file 1 [file sensors-25-06074-s001.zip › Supplementary_files/S1/lstm_135/Network_Cross_Validation/KW_frho135_ncv3.png]

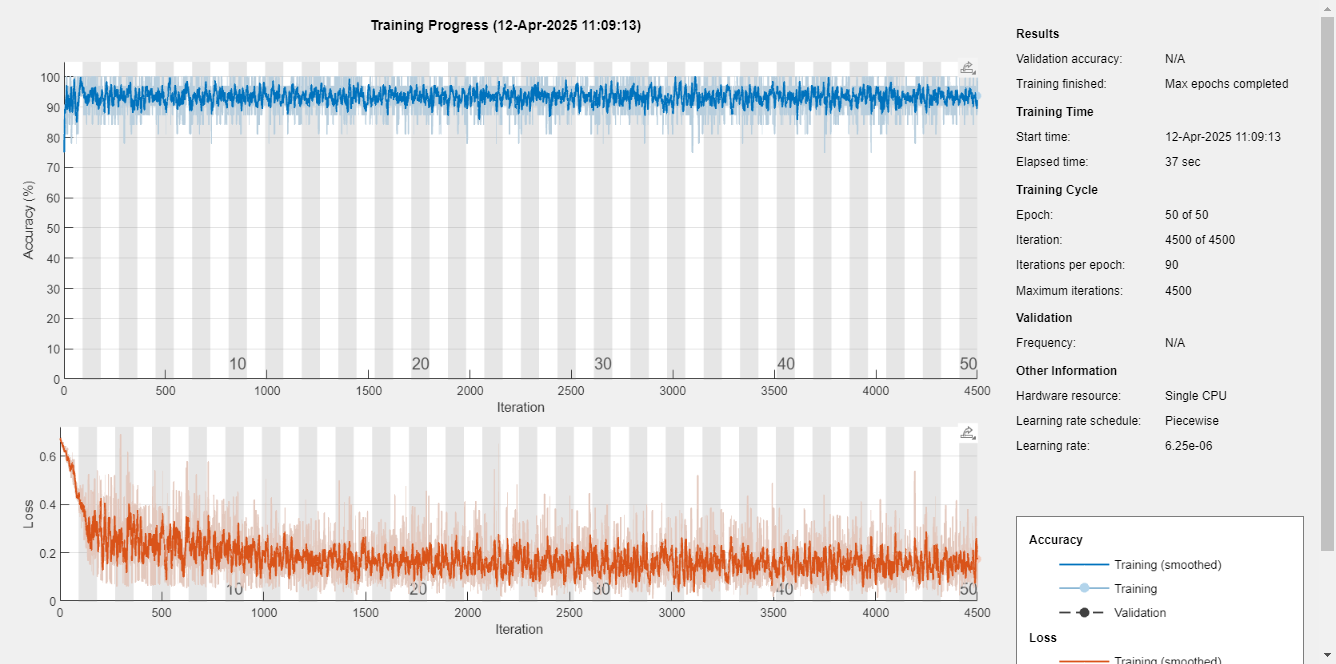

Supplement: Supplementary file 1 [file sensors-25-06074-s001.zip › Supplementary_files/S1/lstm_135/Network_Cross_Validation/KW_frho135_ncv4.png]

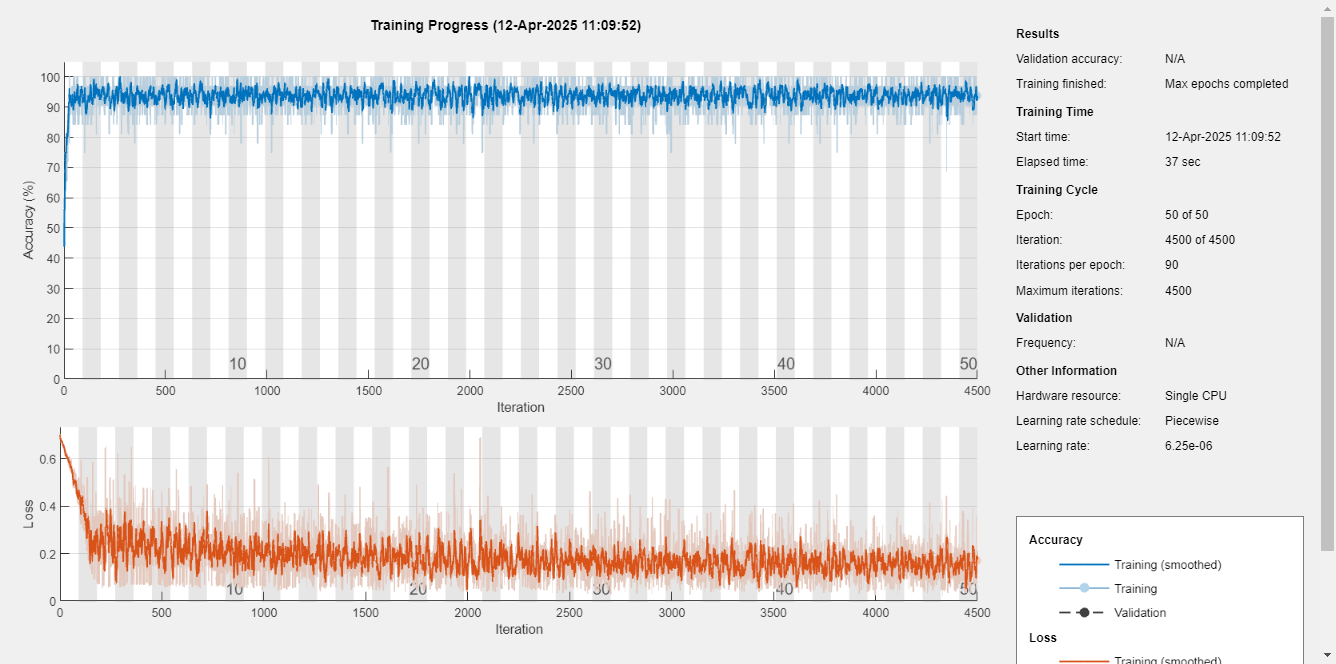

Supplement: Supplementary file 1 [file sensors-25-06074-s001.zip › Supplementary_files/S1/lstm_135/Network_Cross_Validation/KW_frho135_ncv5.png]

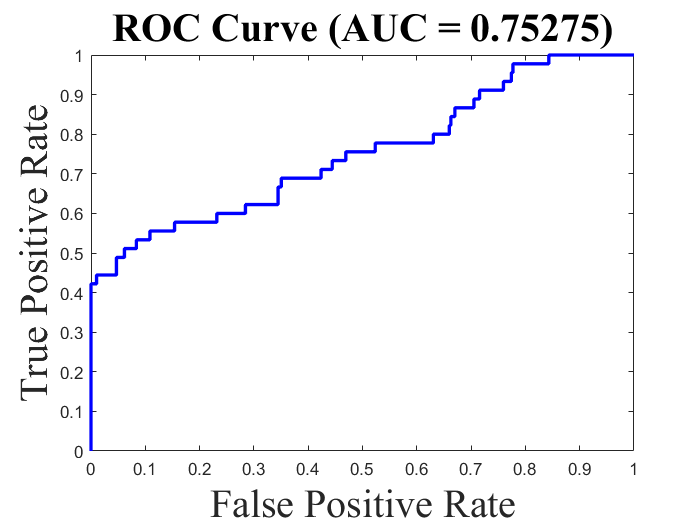

Supplement: Supplementary file 1 [file sensors-25-06074-s001.zip › Supplementary_files/S1/lstm_24/AUC/KW_fphi24_roc1.png]

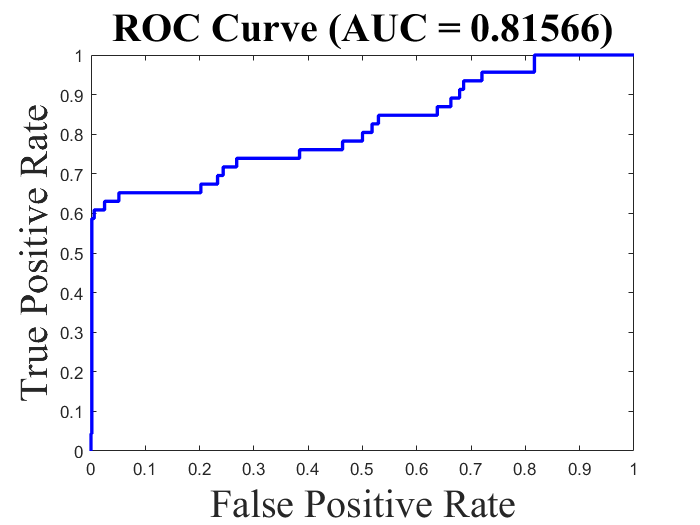

Supplement: Supplementary file 1 [file sensors-25-06074-s001.zip › Supplementary_files/S1/lstm_24/AUC/KW_fphi24_roc2.png]

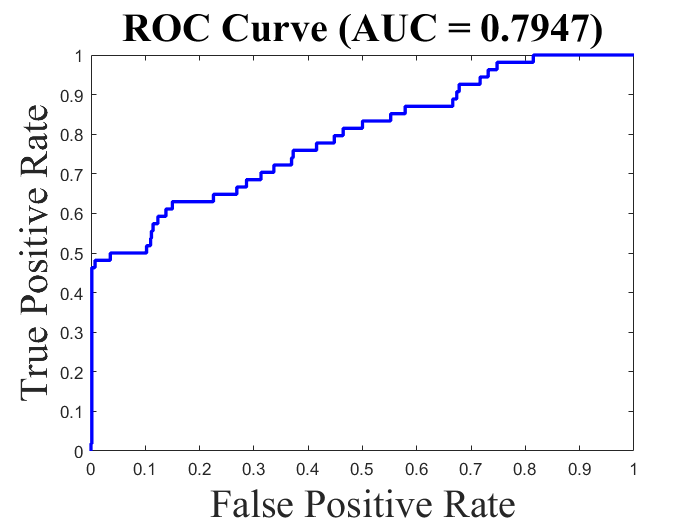

Supplement: Supplementary file 1 [file sensors-25-06074-s001.zip › Supplementary_files/S1/lstm_24/AUC/KW_fphi24_roc3.png]

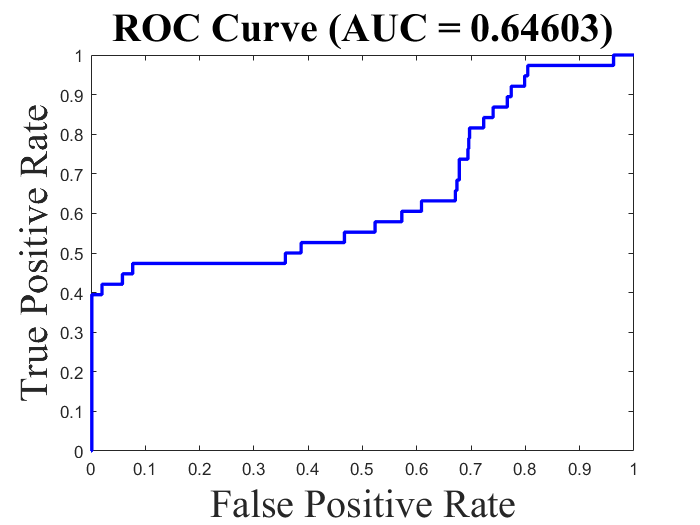

Supplement: Supplementary file 1 [file sensors-25-06074-s001.zip › Supplementary_files/S1/lstm_24/AUC/KW_fphi24_roc4.png]

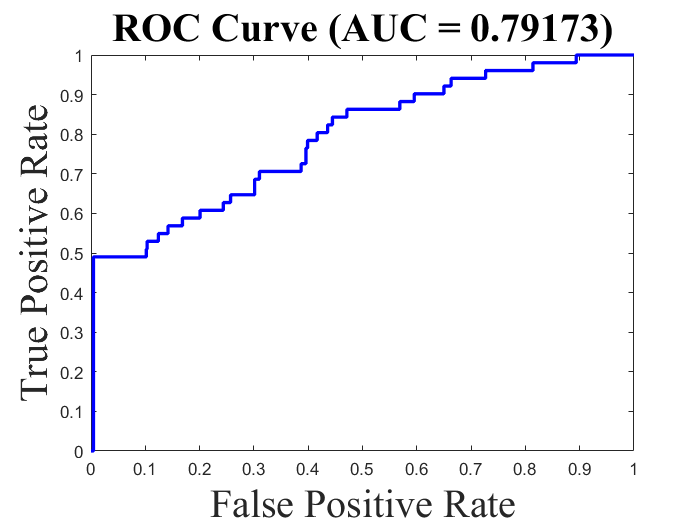

Supplement: Supplementary file 1 [file sensors-25-06074-s001.zip › Supplementary_files/S1/lstm_24/AUC/KW_fphi24_roc5.png]

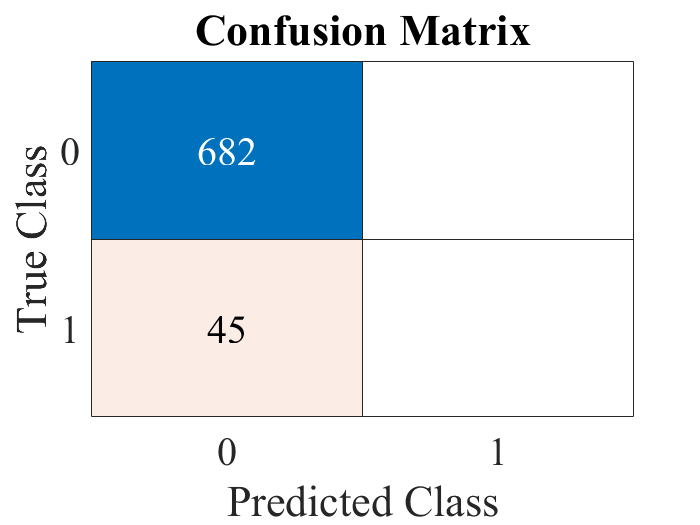

Supplement: Supplementary file 1 [file sensors-25-06074-s001.zip › Supplementary_files/S1/lstm_24/Confusion_matrix/KW_fphi24_cm1.png]

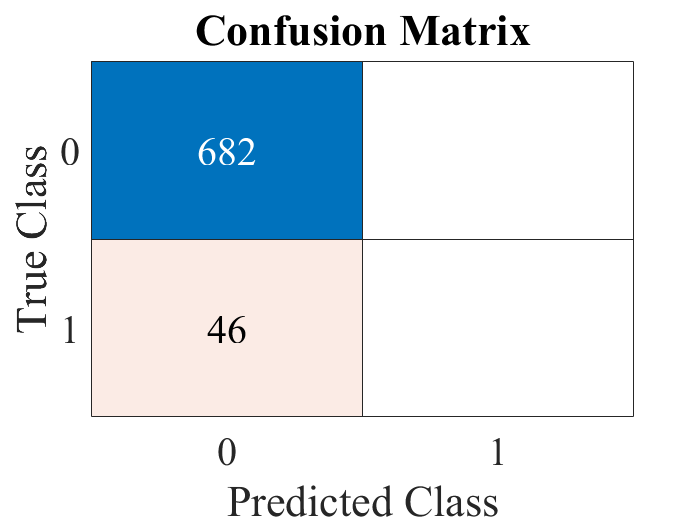

Supplement: Supplementary file 1 [file sensors-25-06074-s001.zip › Supplementary_files/S1/lstm_24/Confusion_matrix/KW_fphi24_cm2.png]

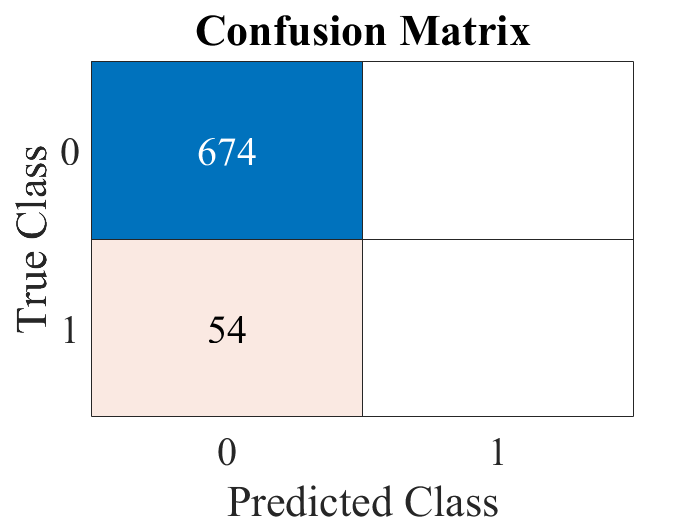

Supplement: Supplementary file 1 [file sensors-25-06074-s001.zip › Supplementary_files/S1/lstm_24/Confusion_matrix/KW_fphi24_cm3.png]

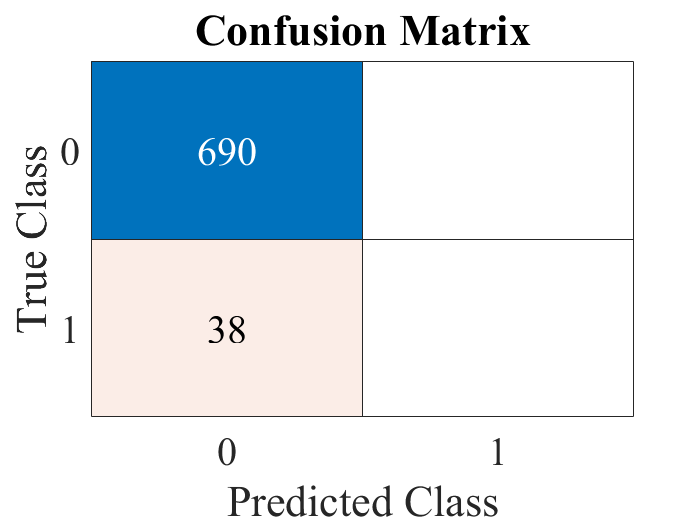

Supplement: Supplementary file 1 [file sensors-25-06074-s001.zip › Supplementary_files/S1/lstm_24/Confusion_matrix/KW_fphi24_cm4.png]

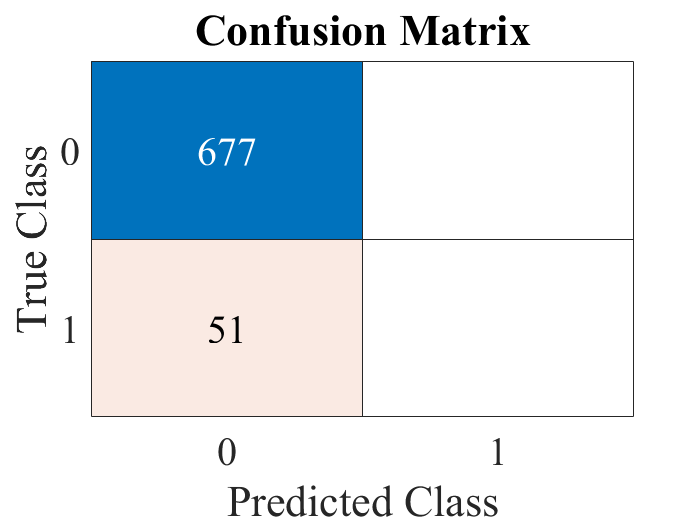

Supplement: Supplementary file 1 [file sensors-25-06074-s001.zip › Supplementary_files/S1/lstm_24/Confusion_matrix/KW_fphi24_cm5.png]

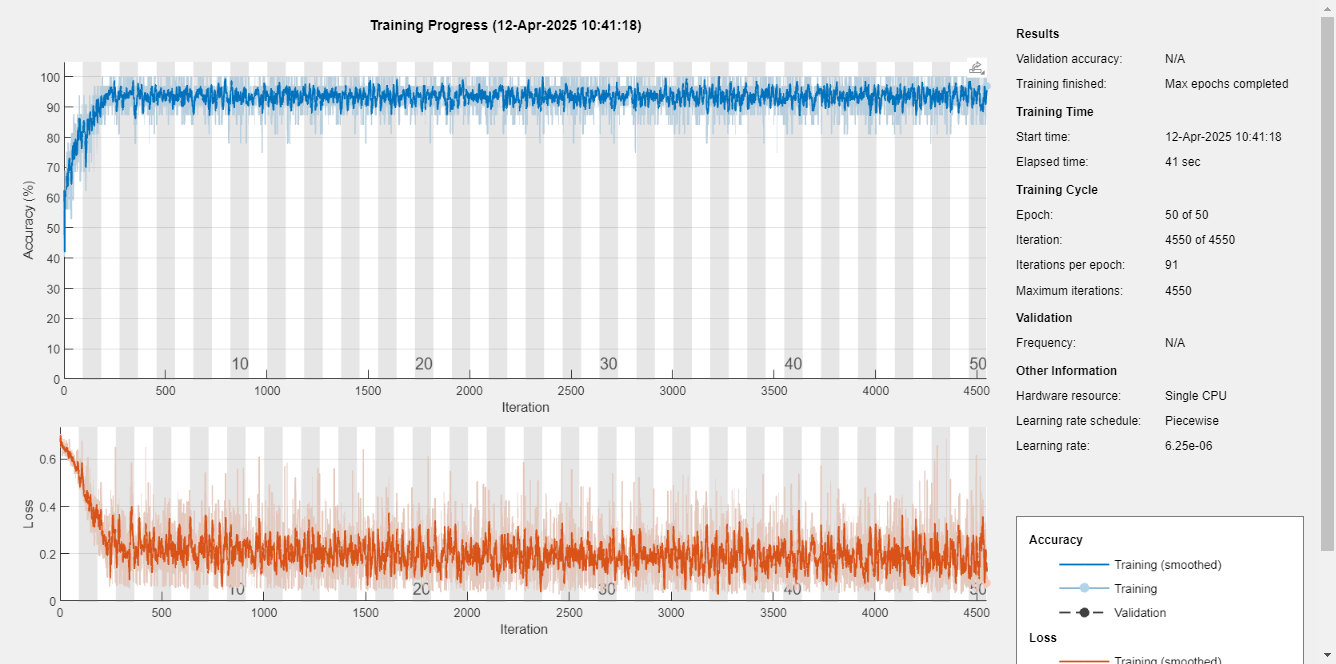

Supplement: Supplementary file 1 [file sensors-25-06074-s001.zip › Supplementary_files/S1/lstm_24/Network_Cross_Validation/KW_fphi24_ncv1.png]

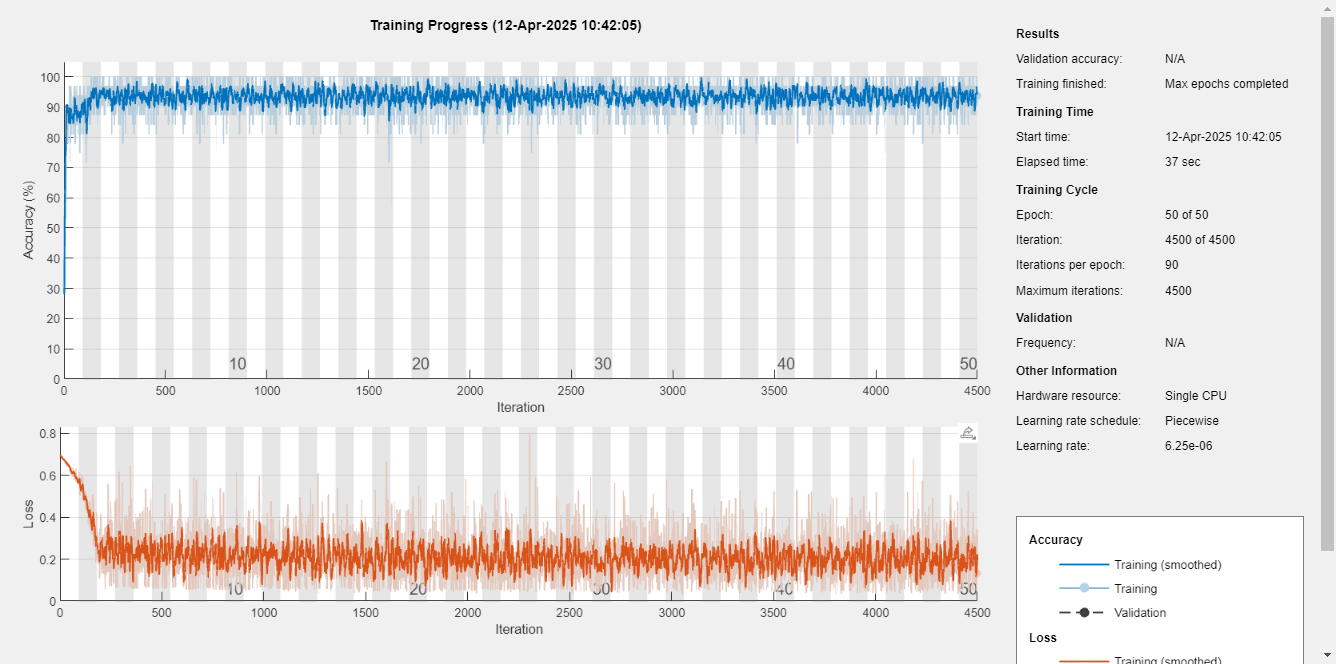

Supplement: Supplementary file 1 [file sensors-25-06074-s001.zip › Supplementary_files/S1/lstm_24/Network_Cross_Validation/KW_fphi24_ncv2.png]

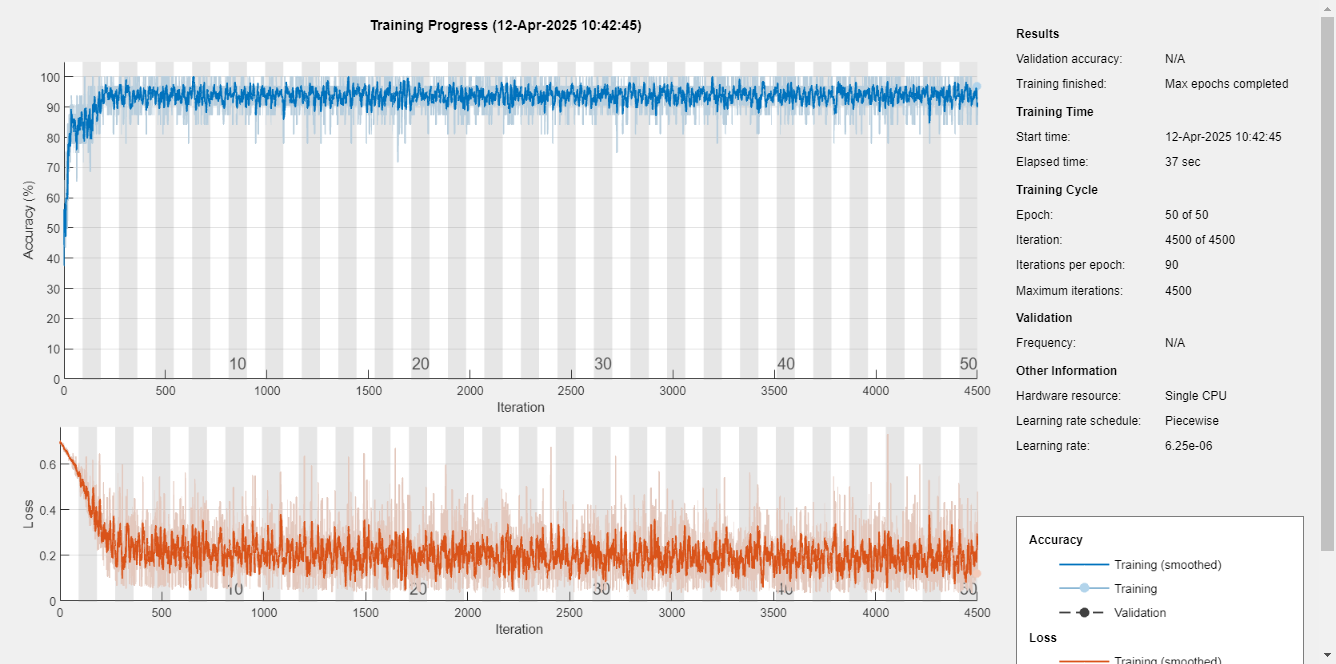

Supplement: Supplementary file 1 [file sensors-25-06074-s001.zip › Supplementary_files/S1/lstm_24/Network_Cross_Validation/KW_fphi24_ncv3.png]

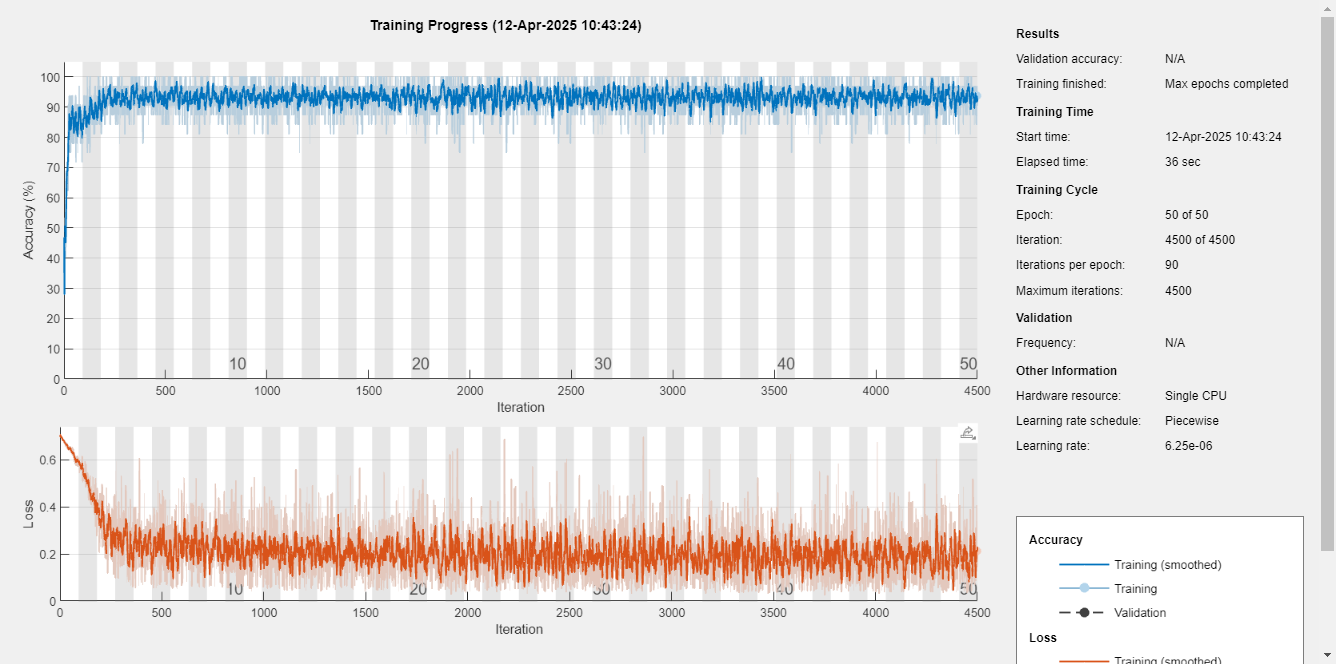

Supplement: Supplementary file 1 [file sensors-25-06074-s001.zip › Supplementary_files/S1/lstm_24/Network_Cross_Validation/KW_fphi24_ncv4.png]

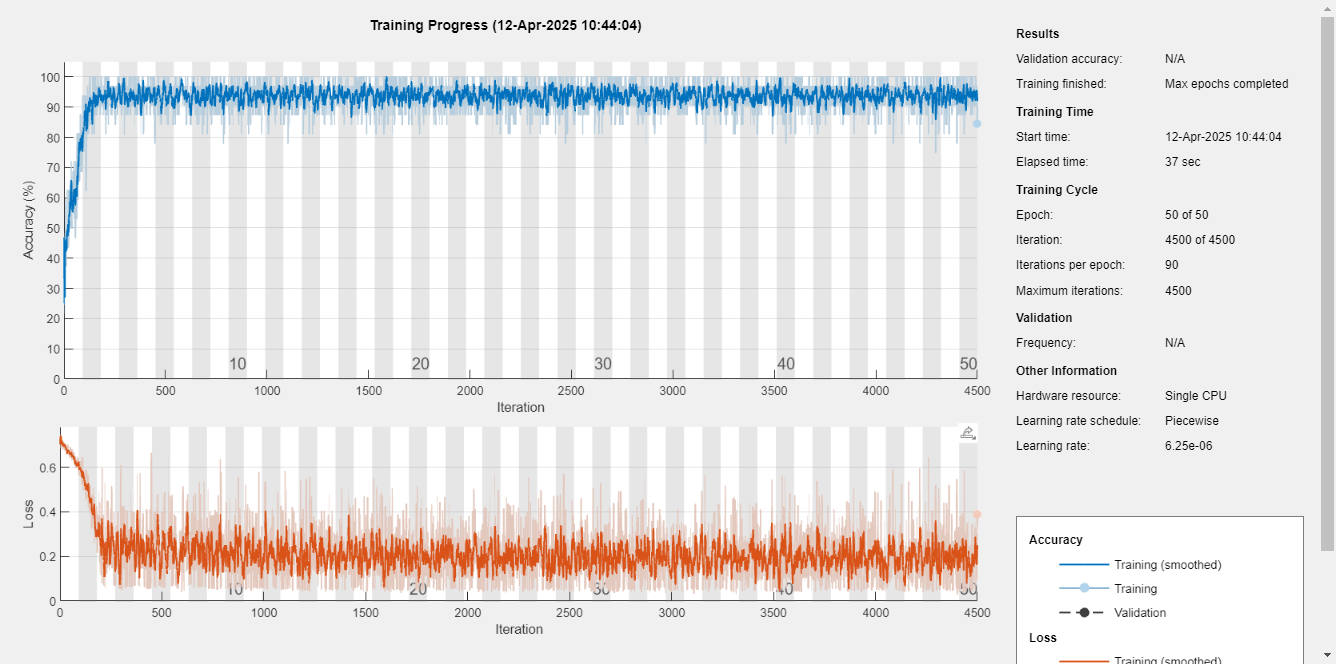

Supplement: Supplementary file 1 [file sensors-25-06074-s001.zip › Supplementary_files/S1/lstm_24/Network_Cross_Validation/KW_fphi24_ncv5.png]

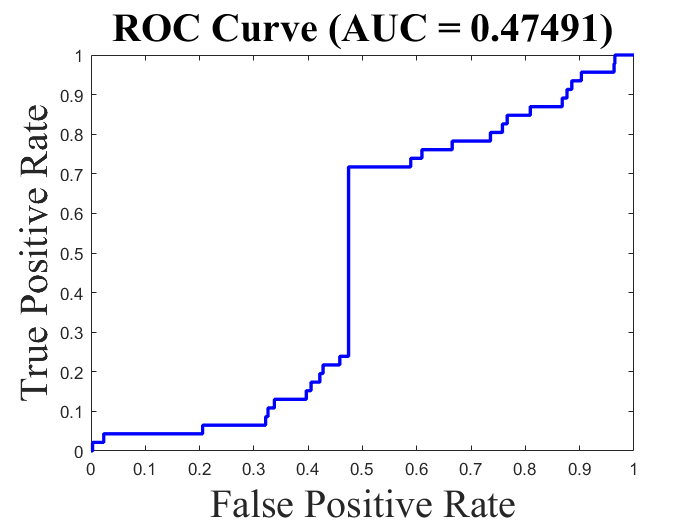

Supplement: Supplementary file 1 [file sensors-25-06074-s001.zip › Supplementary_files/S1/lstm_45/AUC/KW_fcop45_roc1.png]

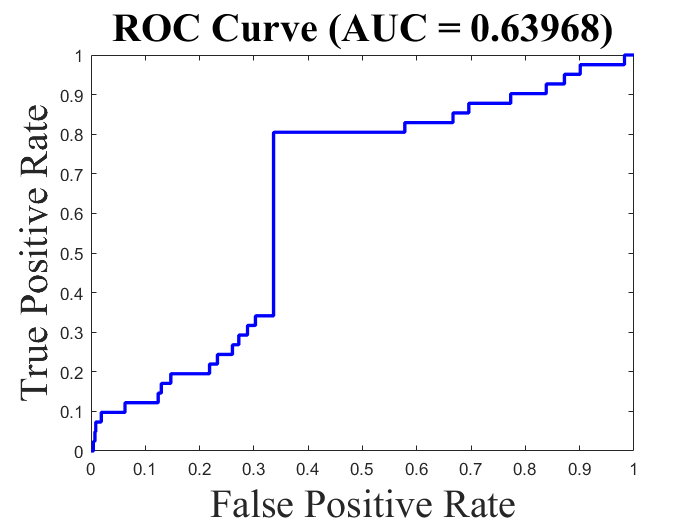

Supplement: Supplementary file 1 [file sensors-25-06074-s001.zip › Supplementary_files/S1/lstm_45/AUC/KW_fcop45_roc2.png]

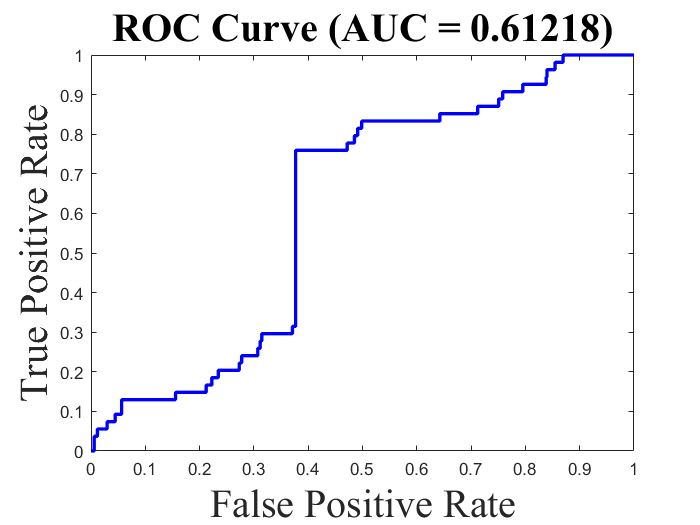

Supplement: Supplementary file 1 [file sensors-25-06074-s001.zip › Supplementary_files/S1/lstm_45/AUC/KW_fcop45_roc3.png]

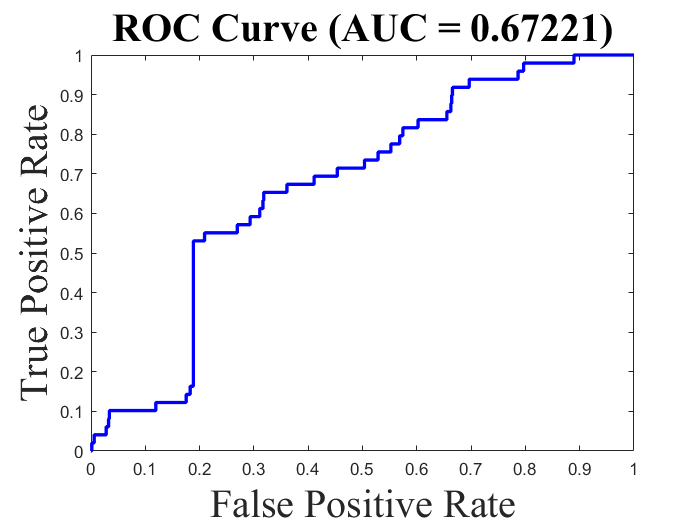

Supplement: Supplementary file 1 [file sensors-25-06074-s001.zip › Supplementary_files/S1/lstm_45/AUC/KW_fcop45_roc4.png]

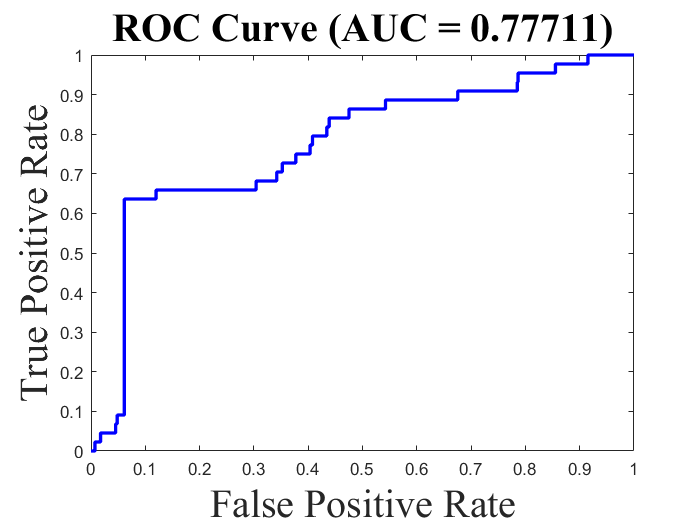

Supplement: Supplementary file 1 [file sensors-25-06074-s001.zip › Supplementary_files/S1/lstm_45/AUC/KW_fcop45_roc5.png]

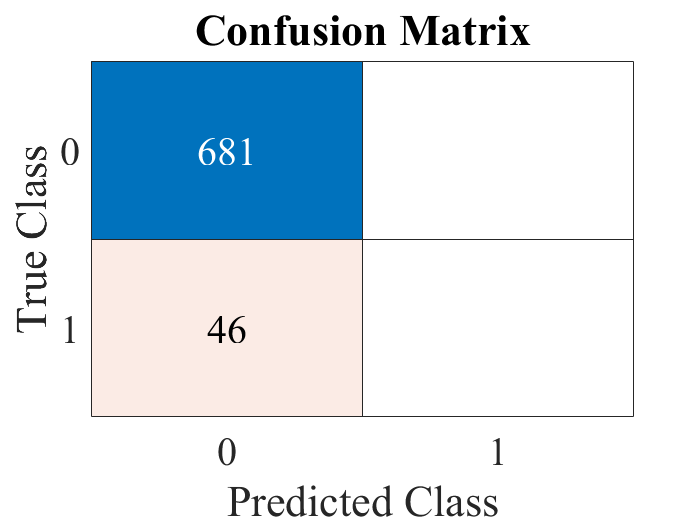

Supplement: Supplementary file 1 [file sensors-25-06074-s001.zip › Supplementary_files/S1/lstm_45/Confusion_Matrix/KW_fcop45_cm1.png]

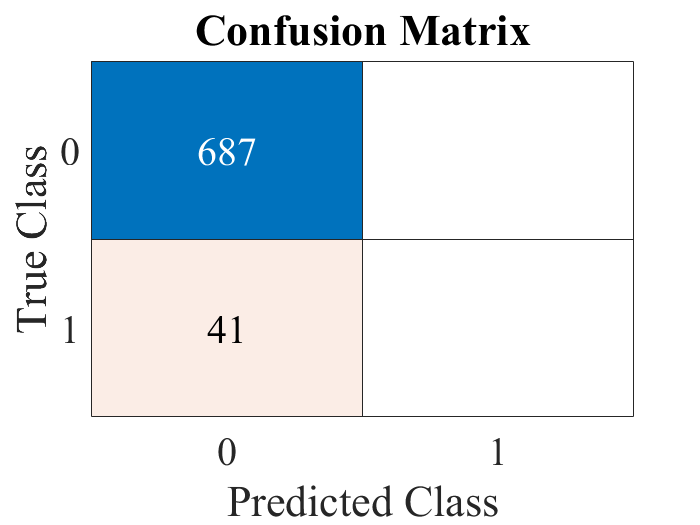

Supplement: Supplementary file 1 [file sensors-25-06074-s001.zip › Supplementary_files/S1/lstm_45/Confusion_Matrix/KW_fcop45_cm2.png]

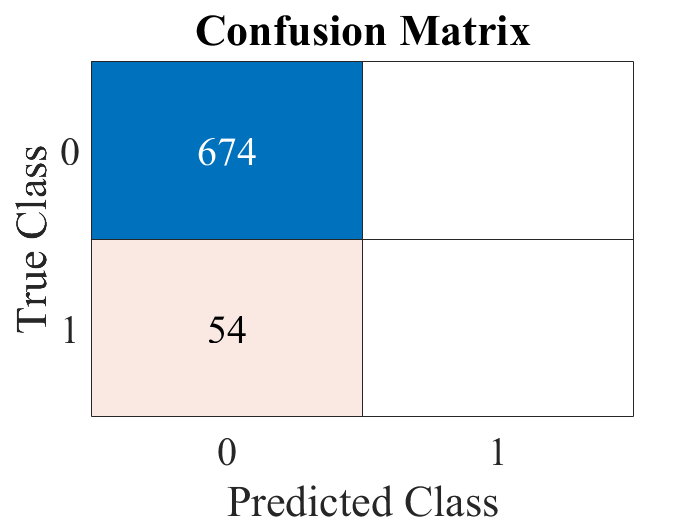

Supplement: Supplementary file 1 [file sensors-25-06074-s001.zip › Supplementary_files/S1/lstm_45/Confusion_Matrix/KW_fcop45_cm3.png]

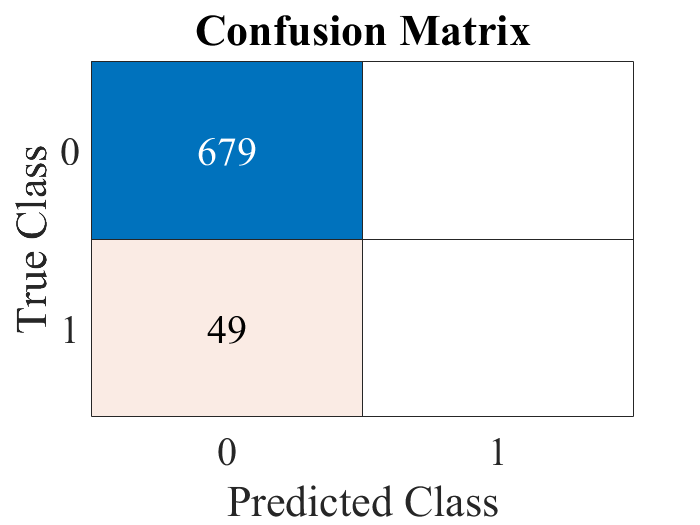

Supplement: Supplementary file 1 [file sensors-25-06074-s001.zip › Supplementary_files/S1/lstm_45/Confusion_Matrix/KW_fcop45_cm4.png]

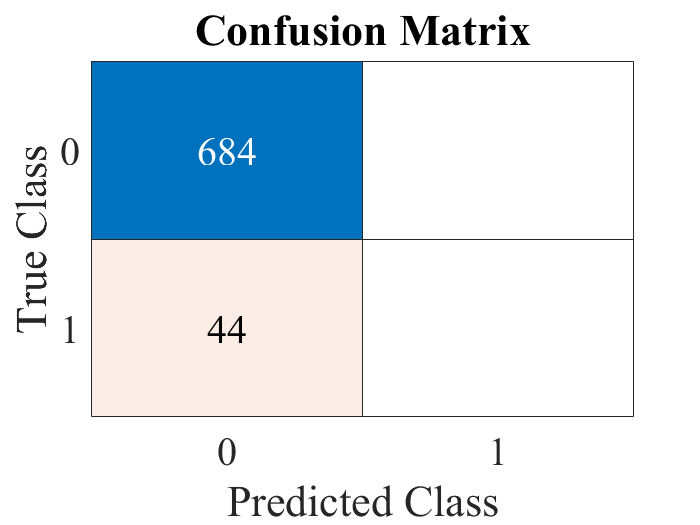

Supplement: Supplementary file 1 [file sensors-25-06074-s001.zip › Supplementary_files/S1/lstm_45/Confusion_Matrix/KW_fcop45_cm5.png]

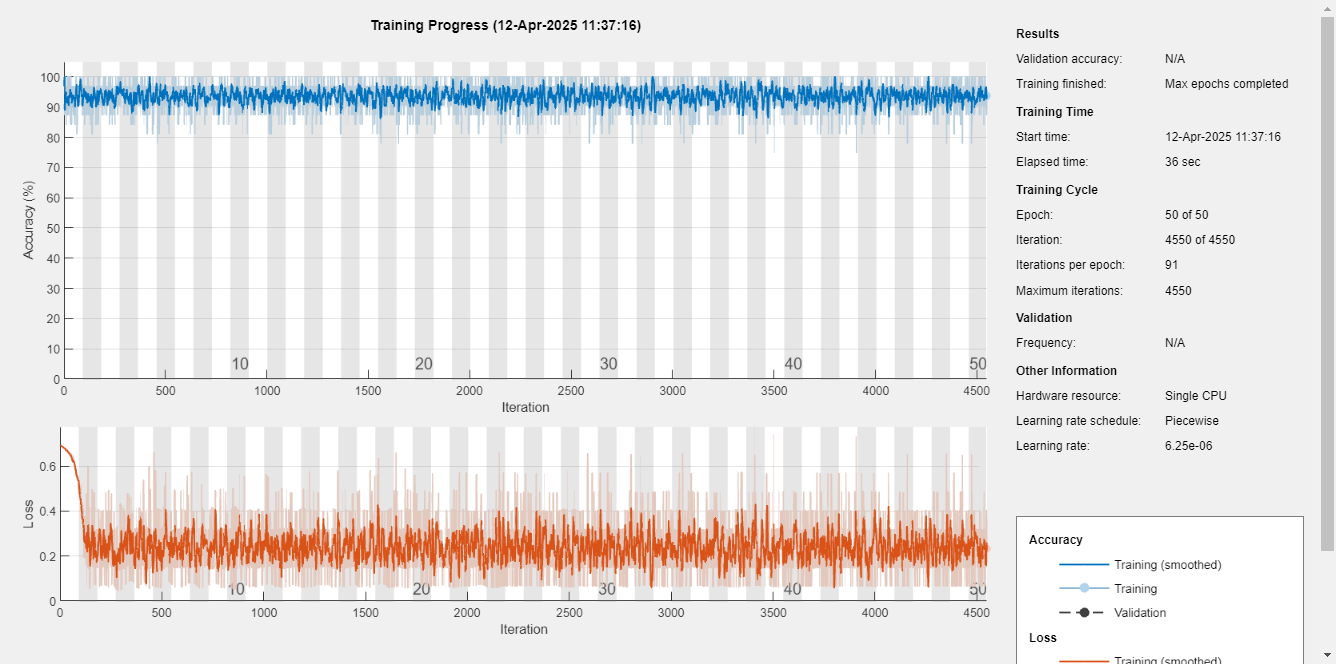

Supplement: Supplementary file 1 [file sensors-25-06074-s001.zip › Supplementary_files/S1/lstm_45/Network_Cross_Validation/KW_fcop45_ncv1.png]

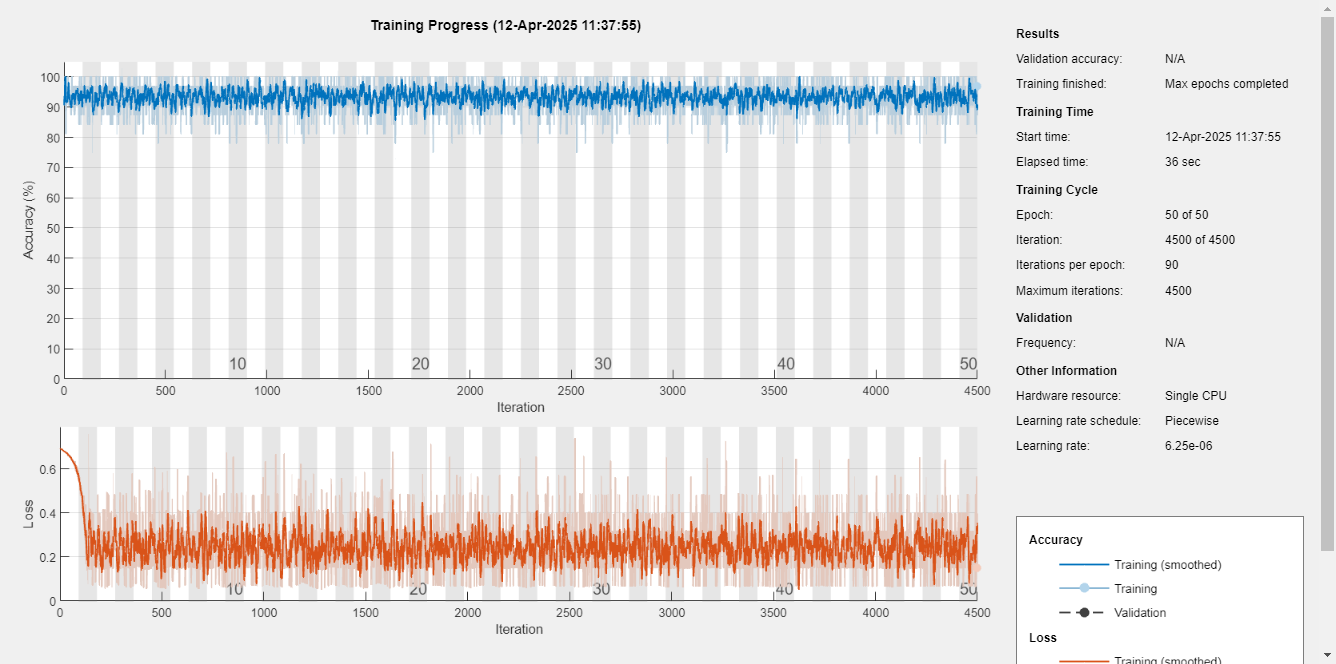

Supplement: Supplementary file 1 [file sensors-25-06074-s001.zip › Supplementary_files/S1/lstm_45/Network_Cross_Validation/KW_fcop45_ncv2.png]

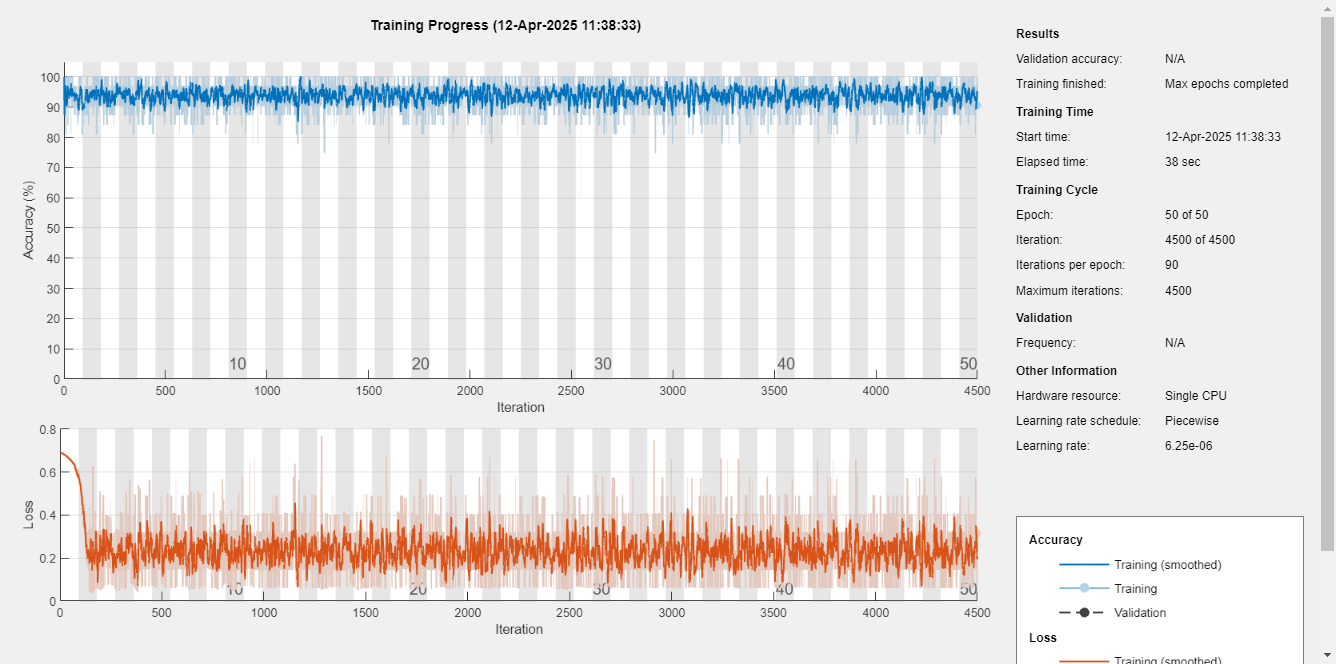

Supplement: Supplementary file 1 [file sensors-25-06074-s001.zip › Supplementary_files/S1/lstm_45/Network_Cross_Validation/KW_fcop45_ncv3.png]

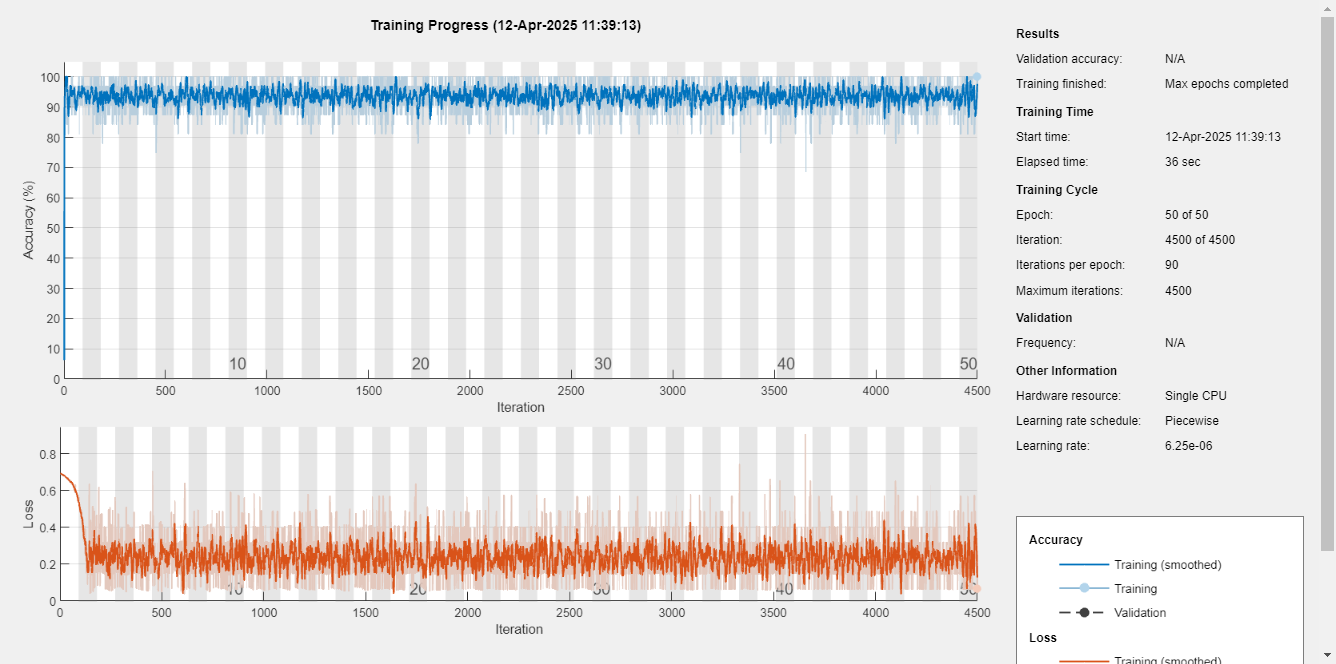

Supplement: Supplementary file 1 [file sensors-25-06074-s001.zip › Supplementary_files/S1/lstm_45/Network_Cross_Validation/KW_fcop45_ncv4.png]

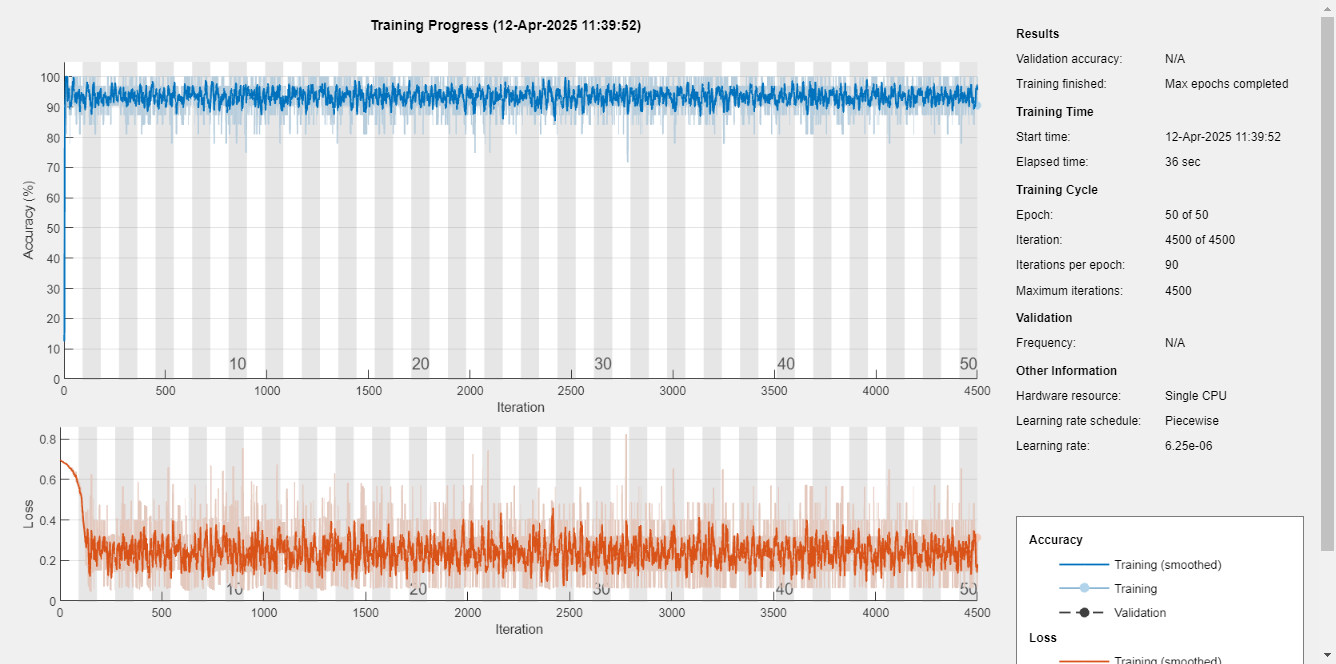

Supplement: Supplementary file 1 [file sensors-25-06074-s001.zip › Supplementary_files/S1/lstm_45/Network_Cross_Validation/KW_fcop45_ncv5.png]

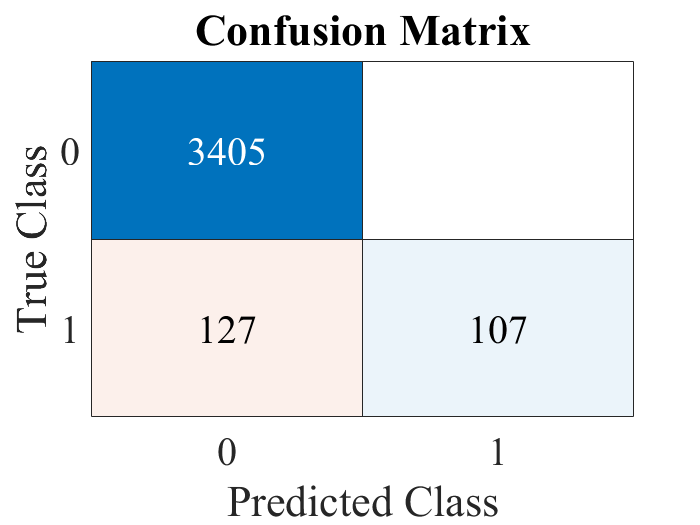

Supplement: Supplementary file 1 [file sensors-25-06074-s001.zip › Supplementary_files/S2/SVM_confusionmatrix/svm123_cm.jpg]

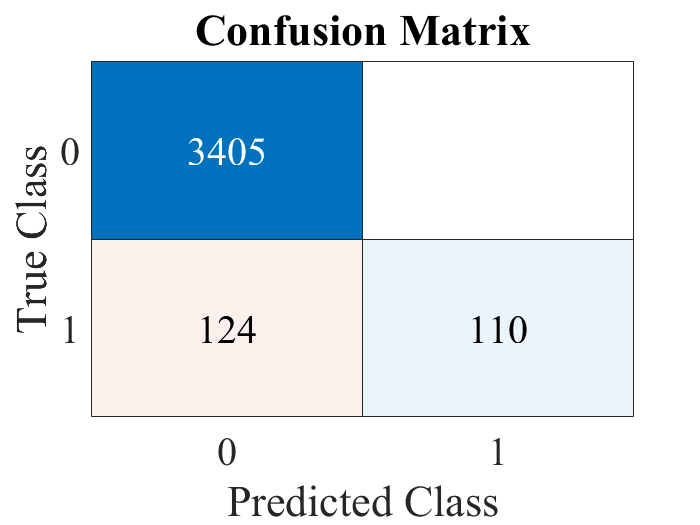

Supplement: Supplementary file 1 [file sensors-25-06074-s001.zip › Supplementary_files/S2/SVM_confusionmatrix/svm12345_cm.png]

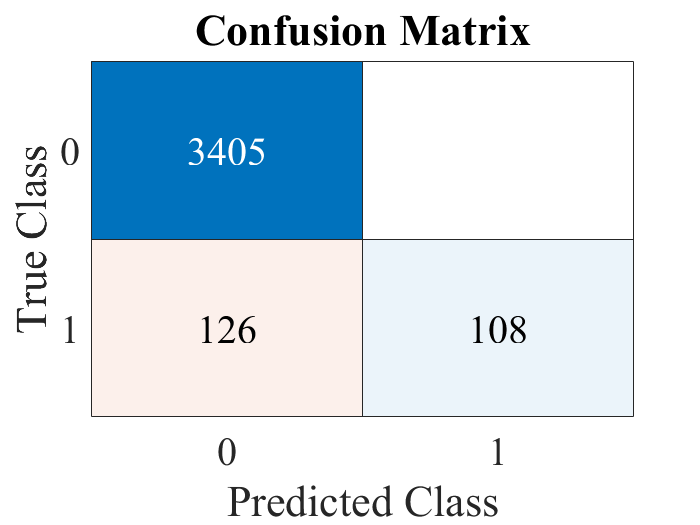

Supplement: Supplementary file 1 [file sensors-25-06074-s001.zip › Supplementary_files/S2/SVM_confusionmatrix/svm135_cm.png]

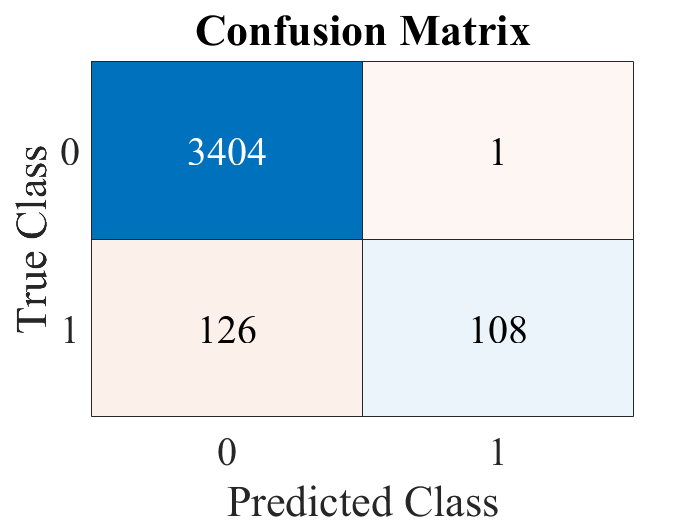

Supplement: Supplementary file 1 [file sensors-25-06074-s001.zip › Supplementary_files/S2/SVM_confusionmatrix/svm24_cm.png]

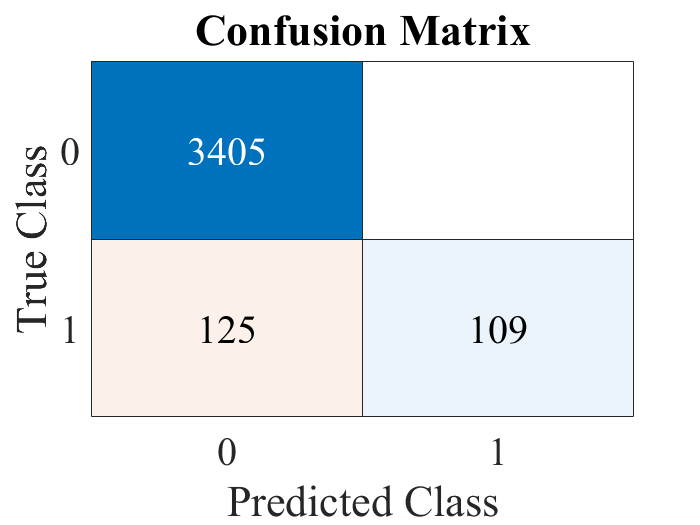

Supplement: Supplementary file 1 [file sensors-25-06074-s001.zip › Supplementary_files/S2/SVM_confusionmatrix/svm45_cm.png]
